# Protein attributes contribute to halo-stability, bioinformatics approach

**DOI:** 10.1186/1746-1448-7-1

**Published:** 2011-05-18

**Authors:** Esmaeil Ebrahimie, Mansour Ebrahimi, Narjes Rahpayma Sarvestani, Mahdi Ebrahimi

**Affiliations:** 1 Department of Crop Production & Plant Breeding, College of Agriculture, Shiraz University, Shiraz, Iran; 2 Bioinformatics Research Group, Green Research Center, Qom University, Qom, Iran; 3 Max-Planck-Institute for Informatics 66123 Saarbrucken, Germany

## Abstract

Halophile proteins can tolerate high salt concentrations. Understanding halophilicity features is the first step toward engineering halostable crops. To this end, we examined protein features contributing to the halo-toleration of halophilic organisms. We compared more than 850 features for halophilic and non-halophilic proteins with various screening, clustering, decision tree, and generalized rule induction models to search for patterns that code for halo-toleration. Up to 251 protein attributes selected by various attribute weighting algorithms as important features contribute to halo-stability; from them 14 attributes selected by 90% of models and the count of hydrogen gained the highest value (1.0) in 70% of attribute weighting models, showing the importance of this attribute in feature selection modeling. The other attributes mostly were the frequencies of di-peptides. No changes were found in the numbers of groups when K-Means and TwoStep clustering modeling were performed on datasets with or without feature selection filtering. Although the depths of induced trees were not high, the accuracies of trees were higher than 94% and the frequency of hydrophobic residues pointed as the most important feature to build trees. The performance evaluation of decision tree models had the same values and the best correctness percentage recorded with the Exhaustive CHAID and CHAID models. We did not find any significant difference in the percent of correctness, performance evaluation, and mean correctness of various decision tree models with or without feature selection. For the first time, we analyzed the performance of different screening, clustering, and decision tree algorithms for discriminating halophilic and non-halophilic proteins and the results showed that amino acid composition can be used to discriminate between halo-tolerant and halo-sensitive proteins.

## Background

An extremophile is an organism that thrives in, and may even require, physically or geochemically extreme conditions that are detrimental to the majority of life on Earth. The archaeal domain contains renowned examples of extremophiles [1]. A small percentage of proteins can tolerate salinity and desiccation stresses. The enzymes from extremely halophilic organisms represent a fascinating example of adaptation because they can per-form their functions *in vivo *and *in vitro *at 4-5 M NaCl, losing activity rapidly when exposed to low salt concentrations [2]. Recently, genes for a number of halophilic enzymes have been cloned, including dihydrofolate reductase from *Haloferax volcanii *[3], glutamate dehydrogenase from *Halobacterium salinarum *[4], and malate dehydrogenase from *Haloarcula marismortui *[5]. Structural features and crystals of some important enzymes in from these organisms such as NAD+-linked opine dehydrogenase[6], glucose dehydrogenase [7], 2Fe-2S ferredoxin [8] and halophilic malate dehydrogenase [9] have been prepared. The molecular mechanisms of halotoleration in these enzymes, however, has not been fully elucidated. Some of the sequenced halophilic enzymes are categorized into the protease family, which contains key enzymes necessary for many critical cellular processes and which are widely used in biotechnology and industrial applications [10]. Information transfer system of archaea has shown gene conservation and differences in the chromosomes and the large extrachromosomal elements among these organisms [11]. Since many members of the archaeal domain are extremophiles, thriving in conditions lethal to most cells, archaea represent an important source of enzymes for applied research and enzymology. For instance, haloarchaea and their enzymes have great potential as biocatalysts in applications requiring low water activity such as reactions with high salt or organic solvent concentrations [12].

Development of laboratory techniques such as site-directed mutagenesis has provided the possibility of molecular breeding of new halophilic enzymes. Addition of amino acid residues at appropriate positions of non-halophilic enzymes can allow for better reversibility from denaturation or functioning at higher salt concentrations [13]. For example, Tokunaga et al., (2008) studied the halophilic characteristics of nucleoside diphosphate kinases (NDKs) from the extremely halophilic archaea, Halobacterium salinarum [13]. Residues 134 and 135 in the carboxy-terminal region of non-halophilic Pseudomonas NDK (PaNDK) consist of an Ala-Ala dipeptide. The double mutation, of A134E-A135E in the C-terminal region of PaNDK changes the Ala-Ala dipeptide to a Glu-Glu dipeptide, conferring halophilic characteristics to this enzyme. An important question in the field is to determine the protein features that are critical for halostability. To address this question, we can use an extremophile enzyme such as halolysin as a mod-el.

A variety of lab techniques have been used to find which protein features are critical for halostability. It has been noted that compared to non-halophilic homologs, halophilic proteins contain a greater proportion of negatively charged amino acids relative to positively charged amino acids [10,13], high GC composition [14] and there is also an increased usage of Glu and Asp [15]. A statistical investigation suggested that the overall hydrophobicity of residues used by halophiles is similar to non-halophiles and the great increase in surface acidic charge of halophilic proteins is the principal mode of halophilic structural stability and effective competition with salt for water [10]. Another study showed that the charged state of two C-terminal region residues (134 and 135) of NDK plays a critical role in determining halophilic characteristics. Changing one dipeptide band was able to affect halostability, which clearly highlights the importance of dipeptide features [13]. These data suggest that the acidic amino acid residues at a particular region, rather than the overall net negative charge, are responsible for halophilicity. It has also been proposed that hydrophobic interactions play an important role in the ability of these proteins to cope with salt stress in a hyper-saline environment [16]. Comparing halophilic dihydrofolate reductase (DHFR) from archaeal *Haloferax volcanii *with non-halophilic DHFRs of other taxa showed that the most profound difference in halophilic DHFRs is a general increase in acidic residues, particularly Asp and Glu, and a decrease in basic residues, particularly Lys. Another striking, and perhaps more important, difference was a dejcrease in the overall hydrophobic content of the halophilic proteins [17]. It has been suggested that halophilic proteins from archaea may maintain their folds in high-salt concentrations by sharing highly negatively charged surfaces and weak hydrophobic cores. It has been shown that a mutant protein Glu243Arg of the malate dehydrogenase was more halophilic and required significantly higher con-centrations of NaCl or KCl for equivalent stability [18].

Halolysin, halophilic alkaline serine protease, has been extracted from archaebacteria such as *Natrialba asiatica*, *Haloferax mediterranei*, *Natrialba magadii *and *Halobacterium sp*. NRC-1 [19]. Halolysin from halophilic archaeon is active at NaCl concentrations of 4-4.5 M and loses its activity at salt concentrations lower than 2 M. This enzyme is a very interesting example of adaptation to harsh conditions [20]. The major limitation of finding important halostability features by laboratory experiments is that examining a large number of features in one experiment is very time-consuming and nearly impossible; to date a comprehensive study on the effects of important features, such as dipeptides, on halostability has not been done. In recent years, the development of bioinformatics tools, such as intelligent data mining and knowledge discovery by artificial neural networks and decision trees, has opened a new window and greatly accelerated research in the field [21].

Bioinformatics and comparative genome analysis are now providing powerful new tools for the molecular dissection of vital phenomena. Recently, different feature selection algorithms, decision tree, and neural networks have been used in finding features that contribute to enzyme thermostability and pH resistance [22] and [23]. For getting more information about new data mining methods such as feature selection algorithms and decision trees and their statistical backgrounds which can be beneficial in illuminating the underlying structure of proteins, please refer to Ebrahimi et al., 2009 [22].

The current study aimed to find the most important features and patterns of halostability contribute to ability to tolerate high salt concentrations. To this end, we applied various modeling techniques to study 894 protein features of halo-tolerant (including halolysin proteins) and halo-sensitive. In addition to statistical analysis, underlying structure of halo-tolerant proteins in comparison with halo-sensitive ones was investigated by different screening, clustering, and decision tree modeling on two datasets with or without feature selection filtering. The findings of this study may provide useful clues for designing halo-resistant proteins.

## Methods

Two hundred and fifty eight halo-tolerant proteins from different organisms including bacteria, fungi, plants, and archaea were extracted from the UniProt Knowledgebase (Swiss-Prot and TrEMBL) and NCBI proteins (Additional file 1). All halolysin sequences (A42605, AAG20619, AAV66536, BAA01049, BAA10958, CAP14928, NP_281139, P29143 and YP_001690274) were present in the halo-tolerant protein group. In addition, six-teen halo-sensitive proteins from bacteria, fungi, and plants were extracted from the UniProt Knowledgebase (Swiss-Prot and TrEMBL) and NCBI.

For each protein sequence, 894 protein features (attributes) such as length, weight, isoelectric point, count and frequency of elements (carbon, nitrogen, sulfur, oxygen and hydrogen), count and frequency of each amino acid, count and frequency of negatively charged, positively charged, hydrophilic and hydrophobic residues, count and frequency of dipeptides, number of α-helices and β-strands, and other secondary protein features were extracted by using various bioinformatics tools and softwares from ExPASy site http://www.expasy.org and CLC bio software (CLC bio, Finlandsgade 10-12, Katrinebjerg 8200 Aarhus N Denmark).

The type of protein (halo-Tolerant, T or halo-Sensitive, S) variable (set based on was set as the output variable and the other variables were set as input variables. All features were classified as continuous variables except the N-terminal amino acid which was classified as categorical. A dataset of these protein features was imported into Clementine software (Clementine_NLV-11.1.0.95; Integral Solution, Ltd.) and RapidMiner software (RapidMiner 5.0.001, Rapid-I GmbH, Stochumer Str. 475, 44227 Dortmund, Germany) for further analysis.

To identify the most important features and find possible patterns contributing to different protein classes, decision tree algorithms were applied to the datasets. These models allowed the development of classification systems that, in their rules, automatically included only the attributes that really matter in making a decision. All data mining models run twice: one time with all features and second time with approved features resulted by feature selection method. During feature selection process, attributes that did not contribute to the accuracy of the tree were ignored; so two datasets, one with whole attributes and the other with just important features were used. The main idea of feature selection was to choose a subset of input variables by eliminating features with little or no predictive information. This process yielded very useful information about the data and could be used to reduce the data to relevant fields before training another learning technique such as a neural network. Various algorithms are available for performing classification and segmentation analysis, and herein we used different decision tree and cluster analysis models. To investigate the effects of the feature selection algorithm on other models' behavior, all models were run with or without feature selection criteria.

## 1. Screening Models

### a. Anomaly detection model

This model was used to identify outliers or unusual cases in the data. Unlike other modeling methods that store rules about unusual cases, anomaly detection models store information on what normal behavior look like. This makes it possible to identify outliers even if they do not conform to any known pattern. While traditional methods of identifying outliers generally examine one or two variables at a time, anomaly detection can examine large numbers of fields to identify clusters or peer groups into which similar records fall. Each record can then be compared to others in its peer group to identify possible anomalies. The further away a case is from the normal center, the more likely it is to be unusual.

### b. Feature selection algorithm

The feature selection algorithm was applied to identify the attributes that have a strong correlation with enzyme halostability. The algorithm considers one attribute at a time to determine how well each predictor alone predicts the target variable. The important value for each variable is then calculated as (1 - *p*), where *p *is the value of the appropriate test of association between the candidate predictor and the target variable. The association test for categorized output variables differs from the test for continuous variables. In our study, when the target value was categorical (as in our datasets), *p *values based on the F statistic were used. The idea was to perform a one-way ANOVA F test for each predictor; otherwise, the *p *value was based on the asymptotic t distribution of a transformation of the Pearson correlation coefficient. Other models, such as likelih-ood-ratio chi-square (which also tests for target-predictor independence), Cramer's V (a measure of association based on Pearson's chi-square statistic), and lambda (a measure of association that reflects the proportional reduction in error when the variable is used to predict the target value) were conducted to check for possible effects of calculation on feature selection criteria. The predictors were then labeled as important, marginal, and unimportant, with values > 0.95, between 0.95-0.90, and < 0.90, respectively.

Various feature selection (or attribute weighting) algorithms were applied to identify the attributes that have strong correlations with output variable (the type of proteins T or S). The models were:

#### Weight by information gain

This operator calculated the relevance of a feature by computing the information gain in class distribution.

#### Weight by information gain ratio

This operator calculated the relevance of a feature by computing the information gain ratio for the class distribution.

#### Weight by rule

This operator calculated the relevance of a feature by computing the error rate of a OneR Model on the dataset without this feature.

#### Weight by deviation

Creates weights from the standard deviations of all attributes. The values can be normalized by the average, the minimum, or the maximum of the attribute.

#### Weight by Chi Squared statistic

This operator calculated the relevance of a feature by computing for each attribute of the input da-taset the value of the chi-squared statistic with respect to the class attribute.

#### Weight by Gini Index

This operator calculated the relevance of an attribute by computing the Gini index of the class dis-tribution.

#### Weight by Uncertainty

This operator calculated the relevance of an attribute by measuring the symmetrical uncertainty with respect to the class. The formulaization for this was:

relevance = 2 * (P(Class) - P(Class | Attribute))/P(Class) + P(Attribute)

#### Weight by Relief

Relief measured the relevance of features by sampling examples and comparing the value of the current feature for the nearest example of the same and of a different class. The resulting weights were normalized into the interval between 0 and 1.

#### Weight by Support Vector Machine (SVM)

Support vector machines (SVMs) are a set of related supervised learning methods that analyze data and recognize patterns, used for classification and regression analysis. In this study, coefficients of the normal vector of a linear SVM were employed as feature weights.

#### Weight by PCA

Used the factors of one of the principal components as feature weights.

## 2. Clustering Models

### a. K-Means

The K-Means model can be used to cluster data into distinct groups when groups are unknown. Un-like most learning methods, K-Means models do not use a target field. This type of learning, with no target field, is called unsupervised learning. Instead of trying to predict an outcome, K-Means tries to uncover patterns in the set of input fields. Records are grouped so that records within a group or cluster tend to be similar to each other, whereas records in different groups are dissimilar. K-Means works by defining a set of starting cluster centers derived from the data. It then assigns each record to the cluster to which it is most similar based on the record's input field values. After all cases have been assigned, the cluster centers are updated to reflect the new set of records assigned to each cluster. The records are then checked again to see whether they should be reassigned to a different cluster and the record assignment/cluster iteration process continues until either the maximum number of iterations is reached or the change between one iteration and the next fails to exceed a specified threshold.

### b. TwoStep cluster

The TwoStep cluster model is a two-step clustering method. The first step makes a single pass through the data, during which it compresses the raw input data into a manageable set of subclusters. The second step uses a hierarchical clustering method to progressively merge subclusters into larger and larger clusters without requiring another pass through the data. Hierarchical clustering has the advantage of not requiring the number of clusters to be selected ahead of time. Many hierarchical clustering methods start with individual records as starting clusters and merge them recursively to produce ever-larger clusters.

## 3. Decision Tree Models

### a. Classification and regression tree (C&RT)

This model uses recursive partitioning to split the training records into segments by minimizing the impurity at each step. A node is considered pure if 100% of cases in the node fall into a specific category of the target field.

### b. CHAID

This method generates decision trees using chi-square statistics to identify optimal splits. Unlike the C&RT and QUEST models, CHAID can generate non-binary trees, meaning that some splits can have more than two branches.

### c. Exhaustive CHAID

This model is a modification of CHAID that does a more thorough job of examining all possible splits, but it takes longer to compute.

### d. QUEST

The QUEST model provides a binary classification method for building decision trees. It is designed to reduce the processing time required for large C&RT analyses while also reducing the tendency found in classification tree methods to favor predictors that allow more splits.

### e. C5.0

The C5.0 model builds either a decision tree or a rule set. The model works by splitting the sample based on the field that provides the maximum information gain at each level. The target field must be categorical. Multiple splits into more than two subgroups are allowed.

## 4. Association Model

The generalized rule induction (GRI) model discovers association rules in the data. GRI extracts a set of rules from the data, pulling out the rules with the highest information content. Information content is measured using an index that takes both the generality (support) and accuracy (confidence) of rules into account.

## 5. One-Way Analysis Of Variance

For each feature, one-way analysis of variance between groups (halolysins, plant proteases, bacterial proteases, fungal proteases, termitases, archaeal non-halophilic) were carried out to determine whether this feature is different between all groups. Pairwise mean comparisons with the Tukey test at p = 0.05 were then carried out by MINITAB 14 to see whether the mean of halolysins for each feature is different from other groups.

## Results

To provide a comprehensive view of structural changes of proteins during halo-stability, for the first time, we tried to cover all aspects of salt-sensitive, salt-tolerant (including halolysin) proteins by extracting and calculating of 894 protein attributes of primary, secondary, and tertiary structures for each sequence. Then, various weighting and modeling algorithms were applied to determine the protein features altering between salt-sensitive, salt-tolerant. Considering a large number of protein features enabled us to detect the key protein characteristics in the structure of salt-tolerant proteins. Some of these features and their descriptive statistics in salt-sensitive, salt-tolerant, and halolysin groups are presented in Additional file 2.

### Feature Selection

When feature selection model was applied on dataset of protein features to compare halo-tolerant with halo-sensitive proteins (T/S groups), 513 of 851 features ranked as important (p > 0.95) implying to contribute to stability to stand in harsh conditions (first hundred features have been presented in Table 1) and 51 features were found to be marginal (0.90 < p > 0.95). A node was generated with just important features and used whenever it was necessary to run all other models on feature selection dataset (as mentioned in Methods). Results of specified feature selection (or attribute weightings) were as follows:

**Table 1:** Results of supervised feature selection on the first 100 important protein attributes (with value equal to 1) contributing to halo-stability of studied proteins.

No	Field	Value	Rank	No	Field	Value	Rank
**1**	Freq of Leu-Leu	1.0	Important	**51**	Freq of Tyr-Leu	1.0	Important

**2**	Freq of Phe-Pro	1.0	Important	**52**	Freq of Glu	1.0	Important

**3**	Freq of Leu-Ile	1.0	Important	**53**	Freq of Tyr-Ile	1.0	Important

**4**	Freq of Glu-Met	1.0	Important	**54**	Freq of carbon	1.0	Important

**5**	Freq of Trp-Lys	1.0	Important	**55**	Freq of Ile-Ile	1.0	Important

**6**	Freq of Leu	1.0	Important	**56**	Freq of Ile-Ala	1.0	Important

**7**	Freq of Val-Phe	1.0	Important	**57**	Freq of Met	1.0	Important

**8**	Freq of Trp-Pro	1.0	Important	**58**	Freq of Other	1.0	Important

**9**	Freq of Lys-Leu	1.0	Important	**59**	Freq of Val	1.0	Important

**10**	Freq of Leu-His	1.0	Important	**60**	Freq of Ala-Lys	1.0	Important

**11**	Freq of Leu-Arg	1.0	Important	**61**	Freq of Arg-Ile	1.0	Important

**12**	Freq of Ile	1.0	Important	**62**	Freq of Ile-Gly	1.0	Important

**13**	Freq of Val-Lys	1.0	Important	**63**	Freq of Lys-Thr	1.0	Important

**14**	Freq of Pro-Tyr	1.0	Important	**64**	Freq of Lys-His	1.0	Important

**15**	Freq of Val-Leu	1.0	Important	**65**	Freq of Phe-Ile	1.0	Important

**16**	Freq of Ser-Phe	1.0	Important	**66**	Freq of sulfur	1.0	Important

**17**	Freq of Val-Gln	1.0	Important	**67**	Freq of Ser-His	1.0	Important

**18**	Freq of Phe-Leu	1.0	Important	**68**	Freq of Lys-Val	1.0	Important

**19**	Freq of Asp-Trp	1.0	Important	**69**	Freq of Leu-Ser	1.0	Important

**20**	Freq of Gly-Leu	1.0	Important	**70**	Freq of His-Ser	1.0	Important

**21**	Freq of Leu-Trp	1.0	Important	**71**	Freq of Ala-Phe	1.0	Important

**22**	Freq of His	1.0	Important	**72**	Freq of nitrogen	1.0	Important

**23**	Freq of Phe	1.0	Important	**73**	Freq of Glu-Ser	1.0	Important

**24**	Freq of Lys	1.0	Important	**74**	Freq of Arg	1.0	Important

**25**	Freq of Tyr-Trp	1.0	Important	**75**	Freq of Met-Gly	1.0	Important

**26**	Freq of Gln-Leu	1.0	Important	**76**	Freq of Ile-Thr	1.0	Important

**27**	Freq of Leu-Val	1.0	Important	**77**	Freq of Pro-Leu	1.0	Important

**28**	Freq of Cys-Tyr	1.0	Important	**78**	Freq of Lys-Ile	1.0	Important

**29**	Freq of Leu-Lys	1.0	Important	**79**	Freq of Try	1.0	Important

**30**	Freq of Met-Leu	1.0	Important	**80**	Freq of Phe-Thr	1.0	Important

**31**	Freq of Thr-Phe	1.0	Important	**81**	Freq of Leu-Pro	1.0	Important

**32**	Freq of Val-Ile	1.0	Important	**82**	Freq of Ile-Val	1.0	Important

**33**	Freq of Leu-Gly	1.0	Important	**83**	Freq of Ile-Trp	1.0	Important

**34**	Freq of Gly-Ile	1.0	Important	**84**	Count of Trp-Lys	1.0	Important

**35**	Freq of Hydrophobic	1.0	Important	**85**	Freq of His-Leu	1.0	Important

**36**	Freq of Tyr-Lys	1.0	Important	**86**	Freq of Gly-Ala	1.0	Important

**37**	Freq of Thr-Val	1.0	Important	**87**	Freq of Ala-Val	1.0	Important

**38**	Freq of Ile-Asn	1.0	Important	**88**	Count of Trp-Pro	1.0	Important

**39**	Freq of His-Gln	1.0	Important	**89**	Freq of Val-Pro	1.0	Important

**40**	Freq of Glu-Gly	1.0	Important	**90**	Freq of Ser-Ile	1.0	Important

**41**	Freq of Leu-Tyr	1.0	Important	**91**	Freq of Glu-Lys	1.0	Important

**42**	Freq of Met-Arg	1.0	Important	**92**	Freq of oxygen	1.0	Important

**43**	Freq of Ala-Leu	1.0	Important	**93**	Freq of Thr-Leu	1.0	Important

**44**	Freq of Gln-Met	1.0	Important	**94**	Freq of Leu-Cys	1.0	Important

**45**	Freq of Trp-Leu	1.0	Important	**95**	Freq of Ile-Leu	1.0	Important

**46**	Freq of Thr-His	1.0	Important	**96**	Freq of Leu-Ala	1.0	Important

**47**	Freq of Ile-Arg	1.0	Important	**97**	Freq of Phe-Val	1.0	Important

**48**	Freq of Pro-Val	1.0	Important	**98**	Freq of Thr-Lys	1.0	Important

**49**	Freq of Tyr-Phe	1.0	Important	**99**	Freq of Tyr	1.0	Important

**50**	Count of hydrogen	1.0		**100**	Freq of Glu-Ala	1.0	Important

The algorithm considers one attribute at a time to determine how well each predictor alone predicts the target variable. The important value for each variable is then cal-culated as (1-p), where p is the p value of the appropriate test of association between the candidate predictor and the target variable. Since the target value was categorical, p values based on the F statistic was used.

#### Weight by information gain

Thirty eight attributes gained weights higher than 0.70 and only the count of hydrogen gained the highest weight (equal to 1.0). The frequencies of Leu - Val and Thr - Val gained weights higher than 0.90 (0.95 and 0.94, respectively). The frequencies of Val, Leu - Arg, Leu, Leu - Ala, Leu - Leu, Gly, Asn, Pro - Val, Lys - Leu, Ala, Trp, Tyr, Val - Ser, hydrophobic residues, Glu - Leu, carbon, oxygen and hydrophilic residues were the next protein attributes weighed in this model (Table 2).

**Table 2:** The most important protein attributes selected by all used attribute weighting algorithms in this study.

Chi Square		Deviation		Gini Index		Uncertainty		Relief		SVM		PCA	
Freq of Cys	1.00	hydrogen	1.00	Count of hydrogen	1.00	Freq of Val-Gln	1.00	Hydrogen	1.00	Freq of Lys-Val	1.00	Count of Ser-Leu	1.00

Freq of Asn	1.00	Freq of Leu-Val	0.95	Freq of Val	0.85	Freq of Ala-Val	0.96	Freq of carbon	0.98	Freq of Val-Ile	0.97	Count of Ile	1.00

Freq of Val	0.99	Freq of Thr-Val	0.95	Freq of Leu-Val	0.81	Freq of Leu-Val	0.95	Freq of oxygen	0.90	Freq of Lys-Cys	0.93	Count of Met	0.93

Freq of Lys	0.95	Freq of Val	0.88	Freq of Thr-Val	0.81	Freq of Thr-Val	0.92	Freq of Hydrophobic Residues	0.89	Freq of Asn-Asn	0.93	Count of Phe	0.92

Freq of Leu-Val	0.94	Freq of Leu-Arg	0.88	Freq of Val-Val	0.79	Freq of Gly-Leu	0.91	Freq of Leu	0.85	Freq of Glu-Met	0.92	Count of Leu-Ser	0.89

Freq of Val-Gln	0.93	Freq of Leu	0.88	Freq of Ala-Tyr	0.78	hydrogen	0.90	Freq of nitrogen	0.83	Freq of Ala-Tyr	0.89	Count of Leucine (L)	0.87

Freq of Gly	0.93	Freq of Leu-Ala	0.86	Freq of Leu	0.78	Freq of carbon	0.90	Freq of Val	0.78	Freq of Lys-Thr	0.88	Count of Tyr	0.83

Freq of Leu-Gly	0.92	Freq of Leu-Leu	0.86	Freq of Ala-Val	0.76	Freq of Thr-Lys	0.89	Freq of Ile	0.74	Freq of Asp-Pro	0.82	Non-reduced Cys Ext coef	0.82

Freq of Leu-Leu	0.92	Freq of Gly	0.86	Freq of Gly	0.76	Freq of Leu-Arg	0.88	Freq of Hydrophilic Res	0.73	Freq of Val-Lys	0.81	Reduced Cys Ext coef	0.82

Freq of Leu-Arg	0.92	Freq of Asn	0.86	Freq of Hydrophobic Residues	0.74	Freq of Leu-Leu	0.88	Freq of Other	0.72	Freq of Thr-Val	0.80	Count of Leu-Phe	0.81

Freq of Ala-Val	0.90	Freq of Pro-Val	0.86	Freq of Gly-Ala	0.74	Freq of Nitrogen	0.88	Freq of Gly	0.67	Freq of Ile-Trp	0.79	Count of Tyr-Lys	0.80

Freq of Hydrophilic Residues	0.89	Freq of Lys-Leu	0.84	Freq of Leu-Leu	0.72	Freq of Val	0.88	Freq of Thr	0.65	Freq of Leu-Leu	0.79	Count of Ile-Leu	0.78

Freq of Val-Lys	0.88	Freq of Ala	0.84	Freq of Ala	0.72	Freq of Thr-His	0.88	Freq of Asp	0.62	Freq of Asn-Gln	0.79	Count of Leu-Tyr	0.77

Freq of Glu-Ala	0.88	Freq of Tryp	0.84	Freq of Leu-Arg	0.71	Freq of Cys	0.88	Freq of His	0.61	Freq of Ser-Cys	0.78	Count of sulfur	0.77

Freq of Thr-Val	0.87	Freq of Tyr	0.84	Freq of Gly-Leu	0.71	Freq of Trp-Pro	0.88	Freq of Phe-Pro	0.61	Freq of Phe-Pro	0.77	Count of Val-Ser	0.72

Freq of Gly-Leu	0.87	Freq of Val-Ser	0.84	Freq of Leu-Gly	0.70	Freq of Asn	0.87	Freq of Val-Ile	0.61	Freq of Gly-Leu	0.77	Count of Ala-Tyr	0.72

Freq of Glu	0.86	Freq of Hydrophobic Residues	0.83	Freq of Val-Lys	0.70	Freq of oxygen	0.87	Freq of Tyr	0.61	Freq of Pro-Arg	0.77	Count of Leu-Leu	0.70

**Information gain**		**Information gain ratio**		**Rule**									
								
Count of hydrogen	1.0	Count of hydrogen	1.0	Freq of hydrophobic residues	1.0								
								
Freq of Leu - Val	0.95	Freq of Glu - Met	0.91	Freq of Ala	1.0								
								
Freq of Thr - Val	0.94	Freq of Gly - Leu	0.91	Freq of hydrogen	o.97								
								
Freq of Val	0.88	Freq of Leu - Leu	0.91	Freq of Gly	0.96								
								
Freq of Leu - Arg	0.87	Freq of Leu	0.91	Freq of Lys	0.96								
								
Freq of Leu	0.86	Freq of Val	0.89	Freq of Asn	0.94								
								
Freq of, Leu - Ala	0.86	Freq of Trp - Pro	0.87	Freq of Val	0.92								
								
Freq of Leu - Leu	0.86	Freq of Ala - Tyr	0.85	Freq of Cys	0.88								
								
Freq of Gly	0.84	Freq of Val - Val	0.80	Freq of Leu	0.87								
								
Freq of Asn	0.84	Freq of Leu - Gly	0.80	Freq of Ser	0.86								
								
Freq of Pro - Val	0.84	Freq of Val - Lys	0.80	Freq of Thr	0.86								
								
Freq of Lys - Leu	0.84			Freq of Leu - Leu	0.85								
								
Freq of Ala	0.84			Freq of hydrophilic residues	0.81								
								
Freq of Trp	0.83												
												
Freq of Tyr	0.81												
												
Freq of Val - Ser	0.81												
												
Freq of hydrophobic residues	0.78												

The figures are the value of each features importance assigned by algorithm.

The frequency of Ala - Tyr, the frequency of Ala - Ala and the count of non - reduced absorption at 289 nm were the most important attributes selected when this model applied on dataset of correlated removed features.

#### Weight by information gain ratio

This operator showed twenty eight features weighed higher than 0.70 and the count of hydrogen, the frequencies of Glu - Met, Gly - Leu, Leu - Leu, Leu, Val, Trp - Pro, Ala - Tyr, Val - Val, Leu - Gly and Val - Lys (with weights of 1.0, 0.91, 0.91, 0.91, 0.91, 0.89, 0.87, 0.85, 0.80, 0.80 and 0.80, respectively) as the most important attributes (Table 2).

When the operator applied to dataset of correlated removed features, the frequency of Ala - Tyr (weight = 1.00), the frequency of Asp - His (weight = 0.86), the count of Gly - Met (weight = 0.81), the count of Trp - Ala (weight = 0.76), the frequency of Ser - Cys (weight = 0.76) and the count of His - Phe (weight = 0.76) gained the highest weights.

#### Weight by rule

Thirty two protein attributes gained weights higher than 0.60 and the frequency of hydrophobic residues and the frequency of Ala obtained the highest weights (1.0). The frequency of hydrogen, the frequency of Gly, the frequency of Lys, the frequency of Asn and the frequency of Val weighed higher than 0.90; while the frequency of other residues, the frequency of Cys, the frequency of Leu, the frequency of Ser, the frequency of Thr, the frequency of Leu - Leu and the frequency of hydrophilic residues gained weights higher than 0.80 (Table 2).

Only two proteins attributes, the percentage of Asn and the frequency of Ala - Tyr with weights of 1.00 and 0.73, chose by rule operator when applied on dataset of correlated removed features.

#### Weight by deviation

Among 38 protein attributes (weight equal to or higher than 0.80) the count of hydrogen, the fre-quencies of Leu - Val, Thr - Val, Val, Leu - Arg, Leu, Leu - Ala, Leu - Leu, Gly and Asn were ten attributes with the highest weights (1.00, 0.95, 0.95, 0.88, 0.88, 0.86, 0.86, 0.86, 0.86 and 0.86).

Just three protein attributes, isoelectric point, the count of Tyr - Val and the count of Ser - Tyr weighing 0.89, 0.88 and 0.84, respectively, highlighted by deviation model when run on dataset of correlated removed features.

#### Weight by Chi Squared statistic

The count of hydrogen gained the highest weight among 39 attributes chose by this model having weights equal to 1.00. The frequencies of Asn, Val, Lys, Leu - Val, Val - Gln, Gly, Leu - Gly, Leu - Leu, Leu - Arg, Ala - Val, hydrophilic residues, Val - Lys, Glu - Ala, Thr - Val, Gly - Leu, Gln, Thr - Lys, Trp, non-educed absorption and reduced absorption attributes with weights between 0.99 and 0.84 were the other next attributes.

The count of non - reduced absorption at 280 nm (weight = 1.00), the frequency of Ala - Tyr (weight = 0.95), the frequency of Ala - Ala (weight 0.73) and the frequency of Ala - Phe (weight = 0.71) were the most important features when Chi Square operator run on dataset of correlated removed features.

#### Weight by Gini Index

Again the count of hydrogen gained the highest weight when this model applied and the weights of frequency of Leu - Val and the frequency of Thr - Val were 0.85 and 0.81. The frequencies of Val - Val, Ala - Tyr, Leu, Ala - Val, Gly, hydrophobic residues, the frequencies of Gly - Ala, Leu - Leu, Ala, Leu - Arg, Gly - Leu, Leu - Gly, Val - Lys, Trp - Lys and Glu - Met were higher than 0.70.

Two attributes (the frequency of Ala - Tyr and the frequency of Ala - Ala weighing 1.00 and 0.91, respectively) selected by this model when correlated removed features dataset used.

#### Weight by Uncertainty

Thirty seven attributes had weights higher than 0.80 with the frequency of Val - Gln having weight equal to 1.00. Four attributes showed weights higher than 0.90 (the frequencies of Ala - Val, Leu - Val, Thr - Val and Gly - Leu) and the count of hydrogen, the frequency of carbon, the frequency of Thr - Lys, the frequency of Leu - Arg, the frequency of Leu - Leu, the frequency of nitrogen, the frequency of Val and the frequency of Thr - His frequencies gained weights of 0.89, 0.89, 0.89, 0.88, 0.88, 0.88, 0.88 and 0.88, respectively.

When the model applied on dataset of correlated removed features, the frequencies of Ala - Tyr, Ala - Ala and Ala - Phe with weights of 1.00, 0.91 and 0.79, respectively, gained the highest weights.

#### Weight by Relief

The count of hydrogen, the frequencies of carbon, oxygen, hydrophobic residues, Leu, nitrogen, Val, Ile, hydrophilic residues, other residues, Gly, Thr, Asp, His, Phe - Pro, Val - Ile and Tyr with weights of 1.00, 0.98, 0.90, 0.89, 0.85, 0.83, 0.78, 0.74, 0.73, 0.72, 0.67, 0.65, 0.62, 0.61, 0.61, 0.61 and 0.60 were seventeen attributes selected by this operator.

Relief operator appointed just two protein attributes (the frequency of Ala - Tyr and the frequency of Ala - Phe with weights of 1.00 and 0.70, respectively) as the most important features when applied on correlated remove features datasets of 156 attributes.

#### Weight by SVM

One attributes (the frequency of Lys - Val), four attributes (the frequency of Val - Ile, the frequency of Lys - Cys, the frequency of Asn - Asn and the frequency of Glu - Met), five attributes (the frequency of Ala - Tyr, the frequency of Lys - Thr, the frequency of Asp - Pro, the frequency of Val - Lys and the frequency of Thr - Val) and nineteen other attributes (Table 2) with weights equal to or higher than 1.00, 0.90, 0.80 and 0.70, respectively, were the features selected by this model.

The frequency of Ala - Tyr (weight equal to 1.00) was the only protein attribute selected by SVM operator when dataset of correlated removed features employed.

#### Weight by PCA

From 18 protein attributes selected by this operator, the count of Ser - Leu had the highest weight (1.00) and then the count of Ile, the count of Met and the count of Phe (with weights of 0.99, 0.93 and 0.92, respectively) and the count of Leu - Ser, the count of Leu, the count of Tyr, non - reduced Cys extinction coefficient, reduced Cys extinction coefficient and the count of Leu - Phe, the count of Tyr - Lys (with weights of 0.89, 0.87, 0.83, 0.82, 0.81 and 0.89, respectively) and the count of Ile - Leu, the count of Leu - Tyr, the count of sulfur, the count of Val - Ser, the count of Ala - Tyr, the count of Leu - Leu and the count of carbon (with weights of 0.78, 0.77, 0.77, 0.72, 0.72, 0.70 and 0.70, respectively) were eighteen attributes selected by this operator.

The count of Ser - Tyr, the count of Tyr - Val, the length of proteins and the count of Phe - Pro with weights of 0.96, 0.87, 0.81 and 0.72 were the most important features appointed by PCA operator when run on dataset of correlated remove features.

Results of attribute weighting models have been presented in Table 2.

### Anomaly detection

When the anomaly detection model was applied on dataset with feature selection criteria, the records divided into two peer groups with an anomaly index cutoff of 5.108, and no record in the first peer group and 2 records in the second peer group (45 and 245 records in groups, respectively) found to be anomaly. The counts of Gly - Ala, Ala and Gly - Ser with average indices of 0.036, 0.026 and 0.037, respectively, occurred in each anomalous record. The same peer groups with the same number of records in each group and the same number of anomalous records found when the model applied on dataset without feature selection, but the count of Ala - Asp, Ala - Gly and Lys - Pro with average indices of 0.015, 0.014 and 0.015, respectively, were the three protein attributes contributed to each ano-malous record.

### K-Means

When K-Means model was applied on database filtered with feature selection, the records were put into 5 groups or clusters (28, 245, 1, 15 and 1 records in each cluster, respectively). When the model was applied on dataset without feature selection filtering, again five clusters with 30, 2, 42, 201 and 15 records were generated. The number of iteration declined from 7 to 3 when feature selection applied on dataset.

### TwoStep model

TwoStep method clustered records into two groups with 45 and 241 records in each cluster, and two clusters (with 45 and 151 records in each cluster) were created for the dataset filtered using feature selection criteria.

### C5.0

The C5.0 model generated a simple decision tree with a depth of 1 and cross-validation of 99.3 ± 0.5. The most important feature used to build the tree was the count of hydrogen. If the value of this feature was ≤ 0.500, the proteins fell into the halo-tolerant category; otherwise, they were put into the halo-sensitive category. The same tree and cross validation values and protein features gained when C5.0 model with 10-fold cross validation applied on dataset. When the same models were applied to datasets using feature selection filtering, a tree with the same depth (1) and cross-validation of 99.0 ± 0.7 and 98.6 ± 0.8 was generated for C5.0 and C5.0 with 10-fold cross-validation, respectively. The count of hydrogen with the same turning point (0.500) again was used to induce the tree

### C&RT

In the C&RT node, a tree with a depth of just 1 was created with the most important feature used to build the tree being the count of hydrogen (value ≤ 0.500 for the T mode and > 0.500 for the S mode). The same results were obtained when feature selection was selected.

### QUEST Model

In QUEST modeling, a tree with a depth of 2 was generated and the frequency of Leu - Leu was used to create the first tree branches. If the value for this attribute was equal to or less than 0.008 and the frequency of Lys - Val was equal to or less than 0.001, the proteins belonged to halo-tolerant (T) group but if the value for the frequency of Lys - Val higher than 0.001, the proteins fell into halo-sensitive (S) group. When the value for the frequency of Leu - Leu was higher than 0.008 and the frequency of Ser - Gln was equal to or less than 0.004, the proteins originated from S group, otherwise from T group. The same results were obtained when feature selection filtering was applied.

### CHAID and Exhaustive CHAID Models

**Figure 1:**
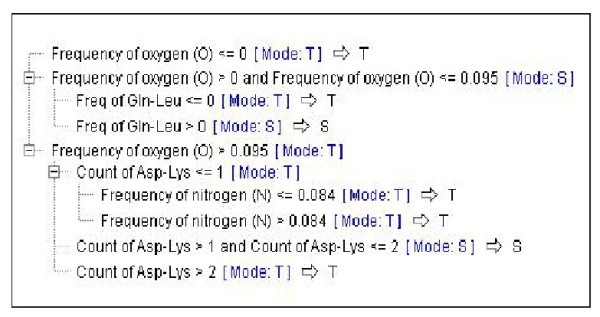
**A decision tree generated by the CHAID modeling method without feature selection filtering comparing halo-tolerant (T) with the halo-sensitive (S) proteins**.

When the CHAID model was applied to the data with or without feature selection, a tree with a depth of 3 was generated. The frequency of oxygen was the main attribute to build the tree branches. If the value for this feature was equal to 0, the protein originated from halo-tolerant (T) group. If the same value was higher than 0 and equal to or less than 0.095 and the frequency of Gln - Leu was equal to 0, the protein fell into T group, otherwise to S group. When the frequency of oxygen was higher than 0.095 and the count of Asp - Lys was higher than 1 and the count of Asp - Lys was equal to or less than 2, the proteins originated from S group, otherwise from T group. The same trees with the same features and values were generated when exhaustive CHAID model applied to datasets without feature selection filtering. When feature selection filtering applied on dataset, again a tree with a depth of 3 generated and the frequency of oxygen with the same values mentioned before used to create tree branches. In addition to the frequency of Gln - Leu, aliphatic index (value of 87.570) and the frequency of Cys - Cys (with turning point of 0) were used to create tree sub-branches. Nearly the same results obtained with exhaustive CHAID model applied on dataset with feature selection filtering (Figure 1).

The best percentage of correctness, performance evaluation, and mean correctness in the decision tree models were observed in the CHAID and Exhaustive CHAID models followed by the C5.0, C5.0 with 10 - fold cross validation, CR&T, CHAID, and finally the QUEST models (Table 3).

**Table 3:** Comparison of percentage of correctness, wrongness, performance evaluation (T & F), and mean correct and incorrect in various decision tree models in datasets with and without feature selection for halo-tolerant and halo-stable protein groups (T/F groups)

Different decision tree models	% Correctness		% Wrongness		Performance evaluation (T)		Performance evaluation (F)		The most important feature (protein attribute) in build the decision tree
		
	Without feature selection	With feature selection	Without feature selection	With feature selection	Without feature selection	With feature selection	Without feature selection	With feature selection	
**C5.0**	99.31	99.31	0.69	0.69	0.053	0.053	2.833	2.833	Count of hydrogen

**C5.0 (10-fold val.)**	99.31	99.31	0.69	0.69	-	0.053	-	2.833	Count of hydrogen

**CR&T**	99.31	98.97	0.61	1.03	-	0.057	-	2.725	Count of hydrogen

**QUEST**	98.97	99.31	1.03	0.69	-	0.053	-	2.833	Frequency of Leu - Leu

**CHAID**	99.66	99.31	0.34	0.69	-	0.053	-	2.833	Frequency of oxygen

**Exhaustive CHAID**	99.66	99.31	0.34	0.69	-	0.053	-	2.833	Frequency of oxygen

### GRI Model

GRI node analysis created 100 rules with 290 valid transactions and minimum and maximum sup-ports of 11.03% and maximum and maximum confidences were 50%. When feature selection was used, minimum support, maximum support, maximum confidence, and minimum confidence changed to 4.83%, 16.9%, 77.55%, and 50.0%, respectively. The highest confidence (77.55%) and support (16.9%) occurred when aliphatic index was > 97.67, but when the length was > 1116, the confidence lowered to 69.57% (Table 4).

**Table 4:** The association rules found in the data by the generalized rule induction (GRI) method, comparing halo-sensitive and halo-tolerant (including halolysin) proteins.

	Antecedent	Support %	Confidence %
1	Aliphatic index > 97.672	16.9	77.55

2	Length > 1116.000	7.93	69.57

3	Aliphatic index > 97.672 and Length > 308.500	10.34	63.33

4	Length > 1116.000 and Length < 1319.500	5.52	56.25

5	Aliphatic index > 97.672 and Non-reduced Cys Extinction coefficient at 280 nm > 19180.000 and the count of Thr> 19.500	8.62	56.0

6	Length > 1116.000 and Aliphatic index > 65.500	5.17	53.33

7	Aliphatic index > 97.672 and Aliphatic index < 105.670	6.55	52.63

8	Aliphatic index > 97.672 and Non-reduced Cys Extinction coefficient at 280 nm > 19180.000 and the count of Ala > 31.500	7.24	52.38

9	Aliphatic index > 97.672 and Length > 308.500 and the count of Ala > 31.500	7.24	52.38

10	Non-reduced Absorption at 280 nm 0.1% (= 1 g/l) > 1.019	7.93	52.17

11	Aliphatic index > 97.672 and Non-reduced Absorption at 280 nm 0.1% (= 1 g/l) > 0.430	7.93	52.17

12	Aliphatic index > 97.672 and Length > 308.500 and the percentage of Try > 0.162	7.93	52.17

13	The count of hydrogen > 0.488	11.03	50.0

14	Aliphatic index > 97.672 and the percentage of His > 1.095	7.59	50.0

15	Aliphatic index > 97.672 and the count of Tyr > 5.500 and the count of Ala > 16.500	7.59	50.0

16	Aliphatic index > 97.672 and the count of Val > 22.500 and the count of Trp-Arg < 1.500	7.59	50.0

17	Aliphatic index > 97.672 and the count of Arg > 10.500 and the count of Ala-Leu > 2.500	7.59	50.0

18	Aliphatic index > 97.672 and the count of Leu > 38.500 and the count of Ala > 16.500	7.59	50.0

19	Aliphatic index > 97.672 and the count of Ile > 18.500 and the count of Ala > 16.500	7.59	50.0

20	Aliphatic index > 97.672 and the count of Gly > 22.500 and the count of Val-Gln > 0.500	7.59	50.0

21	Aliphatic index > 97.672 and the count of neutral charges > 243.000 and the count of Val-Gln > 0.500	7.59	50.0

22	Aliphatic index > 97.672 and the count of hydrophobic residues > 154.500 and the count of Val-Gln > 0.500	7.59	50.0

23	Aliphatic index > 97.672 and the count of oxygen > 460.500 and the count of Val-Gln > 0.500	7.59	50.0

24	Aliphatic index > 97.672 and the count of nitrogen > 398.500 and the count of Val-Gln > 0.500	7.59	50.0

25	Aliphatic index > 97.672 and the count of carbon > 1513.000 and the count of Val-Gln > 0.500	7.59	50.0

26	Aliphatic index > 97.672 and the count of hydrogen > 2430.000 and the count of Val-Gln > 0.500	7.59	50.0

27	Aliphatic index > 97.672 and Reduced Cys extinction coefficient at 280 nm > 18705.000 and the percentage of His > 1.095	7.59	50.0

28	Aliphatic index > 97.672 and Non-reduced Cys extinction coefficient at 280 nm > 19180.000 and the percentage of His > 1.095	7.59	50.0

29	Aliphatic index > 97.672 and Length > 308.500 and the count of Val-Gln > 0.500	7.59	50.0

30	Aliphatic index > 97.672 and Length > 308.500 and the percentage of His > 1.924	6.9	50.0

31	Length > 1116.000 and the count of Ala > 71.500	4.83	50.0

32	Length > 1116.000 and Reduced Cys extinction coefficient at 280 nm > 69840.000 and Aliphatic index > 62.094	4.83	50.0

33	Length > 1116.000 and Non-reduced Cys extinction coefficient at 280 nm > 70530.000 and Aliphatic index > 62.094	4.83	50.0

### One-Way Analysis Of Variance and Mean Comparisons

There was a highly significant difference in 403 of 894 features (p < 0.001 and p < 0. 01) between group of halo-tolerant and halo-sensitive groups. Pairwise comparisons between halo-tolerant and halo-tolerant groups are presented in Additional file 2.

## Discussion

Halodependence and halotolerance are phenotypic characteristics generally included in the "polyphasic" characterization of newly discovered microorganisms toward their description as new taxa with new names and are used to determine their position within the microbial taxonomy [24]. Some use the term for all organisms that require some level of salt for growth, including concentrations around 35 g/L as found in seawater. *Halobacterium *species are obligatory halophilic microorganisms that have adapted to optimal growth under conditions of extremely high salinity. They contain a correspondingly high concentration of salt internally and exhibit a variety of unusual and unique molecular characteristics [25]. Since their discovery, extreme halophiles have been studied extensively by chemists, biochemists, microbiologists, and molecular biologists to understand molecular diversity and universal features of life. A notable list of early research milestones on halophiles includes the discovery of a cell envelope composed of an S-layer glycoprotein [26]. These early discoveries established the value of investigations directed at extremophiles and set the stage for pioneering phylogenetic studies leading to the three-domain view of life and classification of halobacterium as a member of the archaeal domain. It has been shown that some proteins and enzymes are responsible for tolerance to hypersaline conditions [27]. Defining features that contribute to this valuable characteristic can pave the road towards engineering new strains of plants that can grow in harsh salty conditions. To date, some studies have looked at phylogeny, taxonomy and nomenclature of halophilic strains and various models have been employed to determine the most important features that contribute to these organisms' ability to withstand hypersalinity [28]. It should be noted that for future protein engineering applications aimed at converting non-halophilic proteases to halophilic proteases, modification of a small number of amino acids in highly similar sequences will be easier than changing many amino acids [13]. Here, for the first time, we applied different modeling techniques to study more than 850 protein attributes of halophilic proteins (including well-known halolysin proteins) and compared them with halo-sensitive proteins in an attempt to understand the characteristics that allow them to withstand salty conditions. We used different screening, clustering, and decision tree modeling on two datasets with or without feature selection filtering.

The minimum and maximum numbers of protein features (36 and 251) with values higher than 0.50 selected by relief and uncertainty algorithms, respectively. Although the numbers of important protein features in algorithms employed here were different, there were 14 attributes chosen by nearly all algorithms (90% of them) as important features with values higher than 0.50. These features were: the count of hydrogen, the frequencies of carbon, oxygen, hydrophobic and hydrophilic residues, Leu, nitrogen, Val, Phe - Pro, Leu - Gly, Leu - Leu, Leu - Ile, Val - Lys and Ala - Tyr. In 70% of attribute weighting algorithms the count of hydrogen gained the highest possible weight (1.0); showing the importance of this attribute in feature selection modeling.

Although the accuracies of tree induction algorithms were high enough (higher than 94%), the depth of trees generated by the various tree induction models were not high, in the best case the depth reached to two branches and the frequency of hydrophobic residues selected by more than 40% of algorithms as the main protein attribute to build the tree. This feature was also selected as one the most important feature by 90% of attribute weighting algorithms. In this investigation, tree induction models showed the high accuracy in predicting halo-stability condition of a protein based on its structural protein attributes (up to 100% in SVM algorithm). This finding opens a new avenue in protein engineering to predict the halo-stability of an engineered protein before its production. It should be mentioned that because of higher number (replications) of tolerant-proteins in our data set than sensitive proteins, generated models were more precise in prediction of halo-tolerant proteins based on structural characteristics.

A consistent difference exists in the pattern of synonymous codon usage between halophilic and non-halophilic organisms [13] and there is strong evidence that this difference is the result of selection linked to halophilicity [13]. Halophilic and non-halophilic proteins can also be distinguished based on the amino acid composition of their proteomes and several authors have tried to relate these differences to functional adaptation [13]. Significant changes in the frequencies of some amino acids and increases in the their proportions in halophilic organisms (with a two-fold change in the frequency of acidic amino acids) have been documented [29]. In other studies, the residues of some amino acids showed a significant difference (p < 0.01) between mesophilic and thermophilic proteins [30].

The frequency of some di-peptides (Phe - Gly, Leu - Gly, Leu - Leu, Leu - Ile, Val - Lys and Ala - Tyr) were among the most important features in attribute weighting algorithms. To the best of our knowledge, this paper is the first report looked at the effects of all possible di-peptides on halostability. Other previous studies have shown the importance of single acidic amino acid residues (Glu and Asp) [15,31] and Gly [32] in halophilic proteins, here we looked at the importance of dipeptide amino acid composition in salt stability. In another study we showed the frequency of Gln was the most important amino acid in thermostable proteins [23].

Performance evaluations in the decision tree models tested here were found to be the same in all models. No significant differences in the percent of correctness, performance evaluation, and mean correctness of various decision tree models were found when feature selected datasets were used, but positive feature selection effect observed in clustering models.

The number of peer groups (two groups) did not change when feature selection filtering was applied, and also the anomaly index cutoff did not change when feature selection applied on dataset. Although the number of clusters generated by K-Means modeling did not change between the models with or without feature selection, the number of iteration declined from 7 to 3, showing the positive effects of feature selection filtering on removing outliers and the same findings observed with TwoStep modelings.

Charged amino acids prevent charged ions from attaching to proteins and have a significant role in stabilizing proteins against salty conditions and keeping water molecules around these proteins. Feature comparisons showed that, in general, halophilic proteins contained an excess of negatively charged amino acids over positively charged amino acids, and the number of negatively charged amino acid residues was higher than their non-halophilic homologues; this result confirms several previously reported studies [13]. The additional negative charges are primarily located on the protein surface, presumably helping to stabilize the protein molecule by competing with salt for hydra-tion [15]. It has also been proposed that hydrophobic interactions play an important role in the ability of these proteins to cope with the salt stress in a hypersaline environment [16]. It has been shown that negatively charged amino acids, such as Asp and Glu, may contribute to a protein's ability to resist salty conditions; which is consistent with our data that show the presence of a higher percentage of negatively charged amino acid residues (18.5%) in halophilic strains compared to their non-halophilic counterparts [33]. Our findings were also in line with previous studies that showed a higher average of negatively charged amino acids in halolysin proteins compared to other proteins (p < 0.001). It has been shown that the cumulative amount of these amino acids (such as Lys and Arg) and even the content of Val was remarkably high in archaeal salt stable proteins [34]. Higher averages of hydrophobic amino acids found in plant proteins correlates with their function as intracellular proteins that tend to aggregate as a sphere surrounded by water to increase their stability. This may also explain why more positively charged amino acid such as Lys, Arg and His were found in halo-tolerant, although it has been mentioned that this feature may also contribute to salt stability in some organisms [35].

A significant difference (p < 0.05) in aliphatic index was found between halo-tolerant and halo-sensitive proteins, which can be due to the presence of more aliphatic amino acids such as Ile, Val, Pro, Met and Leu in plant proteases. This difference or a higher number of dipeptide bonds may be responsible for the higher number of beta-strands found in halo-tolerant [36].

In recent years, there has been an increasing interest in application of novel bioinformatics ap-proaches in understanding of important phenomena such as salt-tolerance. Paul et al., [37] exploited another bioinformatics approach (comparative genomics and proteomics) to discover different trends between halophiles and non-halophiles. They observed that in the DNA level, the dinucleotide abundance profiles of halophilic genomes keep some common characteristics, which are quite distinct from non-halophiles. These attribute was suggested as the specific genomic signatures for salt-adaptation by this group. At the protein level, halophilic species are characterized by low hydrophobicity, over-representation of acidic residues, especially Asp and underrepresentation of Cys. Our findings are in agreement with Paul group, since in the present study, hydrophobicity, Count of hydrogen, and Asp-related features were highlighted by different weighting and decision tree models as the main distinguishing characteristics to separate halo-tolerant proteins from halo-sensitive ones in different organisms. These findings are promising demonstrating how different bioinformatics methods accords and can be used to address the fundamental question in nature.

## Conclusions

For the first time, we analyzed the performance of different screening, clustering, and decision tree algorithms for discriminating halophilic and non-halophilic proteins. Our results showed that amino acid composition can be used to discriminate between protein groups. We found that most of the mentioned algorithms can be used to discriminate between halophilic and non-halophilic proteins with accuracy in the range of 98-100% and our analysis detected no significant difference in performance between different methods used in this paper. Interestingly, all decision tree models had a similar accuracy (higher than 98%) and no significant differences were observed between analysis with or without feature selection. The best performance and correctness results were obtained with the CHAID tree induction algorithms. In conclusion, we suggest that bioinformatics models can be used as effective tools to discriminate halophilic and non-halophilic proteins. Finally, for the first time we showed the frequency of some di-peptides amino acids can be used as the most important protein attributes to discriminate between halo-tolerant and halo-sensitive proteins.

## Competing interests

The authors declare that they have no competing interests.

## Authors' contributions

NRS prepared protein features, EE initiated the idea and supervised the work while ME and ME did all modeling and prepared the paper draft. All authors read and approved the final manuscript.

## Supplementary Material

Additional file 1 **Accession numbers of protein analyzed in this paper**. This file shows accession numbers of protein extracted from Expasy.org site and analyzed as mentioned in materials and methods. Click here for file

Additional file 2 **Statistical comparison of diverse protein features (attributes) of Salt-sensitive, Salt-tolerant, and Halolysin proteins by T-test**. This file contains results of protein features' statistics. Click here for file
