# Supplementary material for: Protein attributes contribute to halo-stability, bioinformatics approach
Source: Saline Syst. 2011 May 18;7:1. doi: 10.1186/1746-1448-7-1 (PMC3117752; doi:10.1186/1746-1448-7-1)
Supplement: Additional file 1 — Accession numbers of protein analyzed in this paper. This file shows accession numbers of protein extracted from Expasy.org site and analyzed as mentioned in materials and methods. [file 1746-1448-7-1-S1.DOC]

Additional file 1. Accession numbers of proteins studied in this paper

| Accession | Type |
| --- | --- |
| 2DFW|A | T |
| A3LYI0 | T |
| A6ZQG7 | T |
| A6ZRZ0 | T |
| AAA97584 | T |
| AAB35308 | T |
| AAB41279 | T |
| AAD20091 | T |
| AAF45704 | T |
| AAK59503 | T |
| AAK67489 | T |
| AAK76468 | T |
| AAL34180 | T |
| AAL36349 | T |
| AAL85108 | T |
| AAM20327 | T |
| AAN72149 | T |
| AAO42831 | T |
| AAO64157 | T |
| AAP13413 | T |
| AAP68295 | T |
| AAP99840 | T |
| AAS52463 | T |
| AAS52597 | T |
| AAS52864 | T |
| AAT37572 | T |
| AAT37573 | T |
| AAV71142 | T |
| AAY26389 | T |
| ABB16978 | T |
| ABB29467 | T |
| ABF99830 | T |
| ABF99831 | T |
| ABF99832 | T |
| ABI69957 | T |
| ABK24665 | T |
| ABN67640 | T |
| ABR26138 | T |
| ABU88101 | T |
| ABV89657 | T |
| ACG28322 | T |
| ACG33320 | T |
| ACJ76842 | T |
| ACJ76843 | T |
| ACJ76844 | T |
| ACO57636 | T |
| ACO59959 | T |
| ACU12847 | T |
| ACY54022 | T |
| ADB25060 | T |
| BAC57372 | T |
| BAC61752 | T |
| BAD16114 | T |
| BAD19328 | T |
| BAD37282 | T |
| BAE98703 | T |
| BAF00483 | T |
| BAH19748 | T |
| BAH19880 | T |
| BAH20315 | T |
| BAH30374 | T |
| BAH56939 | T |
| BAH56965 | T |
| BAH57083 | T |
| BAH57103 | T |
| BAH57166 | T |
| CAA53245 | T |
| CAA99218 | T |
| CAB78154 | T |
| CAC27336 | T |
| CAC85227 | T |
| CAC85228 | T |
| CAC85244 | T |
| CAC85245 | T |
| CAC85246 | T |
| CAC85247 | T |
| CAG59236 | T |
| CAG60523 | T |
| CAK23824 | T |
| CAK28282 | T |
| CAR21772 | T |
| CAR22217 | T |
| CAR23670 | T |
| CAR26236 | T |
| CAX43089 | T |
| CAY67806 | T |
| CAY68231 | T |
| CAY69181 | T |
| CAY86201 | T |
| CAY86225 | T |
| CAY86961 | T |
| CBI23137 | T |
| DAA07486 | T |
| DAA10695 | T |
| DAA10719 | T |
| DAA10810 | T |
| DAA11432 | T |
| DAA11888 | T |
| EAK94936 | T |
| EAK94942 | T |
| EAK95553 | T |
| EAK95554 | T |
| EAK95556 | T |
| EAK95689 | T |
| EAK95690 | T |
| EAK95694 | T |
| EAK96980 | T |
| EAK97040 | T |
| EAL03099 | T |
| EAL03264 | T |
| EAS78439 | T |
| EAZ63744 | T |
| EDK46503 | T |
| EDN62722 | T |
| EDN63897 | T |
| EDO16019 | T |
| EDP28563 | T |
| EED92441 | T |
| EEF28243 | T |
| EEF32937 | T |
| EEF46831 | T |
| EEF49474 | T |
| EEF49499 | T |
| EEF52662 | T |
| EER34036 | T |
| EER34141 | T |
| EER34142 | T |
| EER34143 | T |
| EER34144 | T |
| EER34145 | T |
| EEU06466 | T |
| EEU06904 | T |
| EEU08365 | T |
| EEX93723 | T |
| NP_001031751 | T |
| NP_001078110 | T |
| NP_001119079 | T |
| NP_001149477 | T |
| NP_001150747 | T |
| NP_001151151 | T |
| NP_001151723 | T |
| NP_009934 | T |
| NP_010324 | T |
| NP_010325 | T |
| NP_013696 | T |
| NP_014552 | T |
| NP_014577 | T |
| NP_014671 | T |
| NP_015330 | T |
| NP_067589 | T |
| NP_172094 | T |
| NP_172527 | T |
| NP_172918 | T |
| NP_174094 | T |
| NP_176629 | T |
| NP_177686 | T |
| NP_178307 | T |
| NP_179760 | T |
| NP_187154 | T |
| NP_187566 | T |
| NP_187842 | T |
| NP_191112 | T |
| NP_192051 | T |
| NP_194171 | T |
| NP_195618 | T |
| NP_196054 | T |
| NP_197815 | T |
| NP_198067 | T |
| NP_198391 | T |
| NP_200358 | T |
| NP_446372 | T |
| NP_524864 | T |
| NP_563717 | T |
| NP_564556 | T |
| NP_565183 | T |
| NP_567030 | T |
| NP_567354 | T |
| NP_799919 | T |
| NP_849598 | T |
| NP_849715 | T |
| NP_850017 | T |
| NP_973802 | T |
| NP_973923 | T |
| O14019 | T |
| O94505 | T |
| P13587 | T |
| P25333 | T |
| P32179 | T |
| P36024 | T |
| P38970 | T |
| P40917 | T |
| P46594 | T |
| P53935 | T |
| P94063.2 | T |
| Q01766 | T |
| Q12180 | T |
| Q12600 | T |
| Q38945 | T |
| Q55034 | T |
| Q59XQ1 | T |
| Q5A2K0 | T |
| Q68KI4 | T |
| Q84TI7 | T |
| Q96288 | T |
| Q9C7A2 | T |
| Q9LDI3 | T |
| Q9LKW9 | T |
| Q9LQZ7 | T |
| Q9LRR7 | T |
| Q9SID1 | T |
| Q9SWE5 | T |
| Q9SYM2 | T |
| XP_001385669 | T |
| XP_001387767 | T |
| XP_001394660 | T |
| XP_001523871 | T |
| XP_001820395 | T |
| XP_001902589 | T |
| XP_002265766 | T |
| XP_002290689 | T |
| XP_002292914 | T |
| XP_002419494 | T |
| XP_002490087 | T |
| XP_002490512 | T |
| XP_002491461 | T |
| XP_002510475 | T |
| XP_002512971 | T |
| XP_002512996 | T |
| XP_002515382 | T |
| XP_002529471 | T |
| XP_002534139 | T |
| XP_002548662 | T |
| XP_002548663 | T |
| XP_002548664 | T |
| XP_002548665 | T |
| XP_002548666 | T |
| XP_002552655 | T |
| XP_002554107 | T |
| XP_714033 | T |
| XP_714604 | T |
| XP_714605 | T |
| XP_714607 | T |
| XP_714735 | T |
| XP_714736 | T |
| XP_714738 | T |
| XP_714739 | T |
| XP_715989 | T |
| XP_716048 | T |
| XP_721878 | T |
| XP_722039 | T |
| YP_001225121 | T |
| YP_001227635 | T |
| YP_003288487 | T |
| YP_748795 | T |
| ZP_01258629 | T |
| ZP_05776413 | T |
| ZP_05905206 | T |
| ZP_05943436 | T |
| A3LPL0 | S |
| A7LIU7 | S |
| B1PRJ8 | S |
| C0LSJ7 | S |
| C6FE28 | S |
| C7SI10 | S |
| CBI26761 | S |
| D0V1M4 | S |
| DAA08195 | S |
| O81223 | S |
| P53260 | S |
| Q2PIT0 | S |
| Q2UMX4 | S |
| Q3YL57 | S |
| Q8W1X2 | S |
| Q9SNC3 | S |
