# Supplementary material for: Protein attributes contribute to halo-stability, bioinformatics approach
Source: Saline Syst. 2011 May 18;7:1. doi: 10.1186/1746-1448-7-1 (PMC3117752; doi:10.1186/1746-1448-7-1)
Supplement: Additional file 2 — Statistical comparison of diverse protein features (attributes) of Salt-sensitive, Salt-tolerant, and Halolysin proteins by T-test. This file contains results of protein features' statistics. [file 1746-1448-7-1-S2.DOC]

**Additional file 2.** Statistical comparison of diverse protein features (attributes) of Salt-sensitive, Salt-tolerant, and Halolysin proteins by T-test. NS: no significant difference, 0.05: significant difference at p=0.05, 0.01: significant difference at p=0.01, and 0.001: significant difference at p=0.001.

| **Protein features (attributes)** | **Salt-sensitive proteins** | **Salt-tolerant proteins** | **Halolysin proteins** | **P-value of comparing Salt-sensitive vs Salt-tolerant** | **P-value of comparing Salt-sensitive vs Halolysin** | **P-value of comparing Salt-tolerant vs Halolysin** |
| --- | --- | --- | --- | --- | --- | --- |
|  | **Mean ± StDev** | **Mean ± StDev** | **Mean ± StDev** |  |  |  |
| **Length** | 719 ± 420 | 479.7 ± 359.6 | 515.8 ± 127.0 | 0.05 | NS | NS |
| **Weight** | 79.4 ± 46.6 | 341.5 ± 1299.5 | 53.25± 11.98 | NS | NS | NS |
| **Isoelectric point** | 6.606 ± 1.347 | 6.577 ± 1.730 | 4.4344 ± 0.2801 | NS | 0.001 | 0.001 |
| **Aliphatic index** | 97.40 ± 10.68 | 82.09 ± 23.04 | 72.90 ± 6.49 | 0.05 | 0.01 | NS |
| **Non-reduced cysteinesExtinctioncoefficient at 280nm** | 84333 ± 58501 | 39823 ± 31894 | 57680 ± 21894 | 0.001 | NS | NS |
| **Non-reduced Absorption at 280nm 0.1% (=1 g/l)** | 0.9835 ± 0.2736 | 0.2409± 0.4542 | 1.0501± 0.2297 | 0.001 | NS | 0.001 |
| **Reduced cysteinesExtinctioncoefficient at 280nm** | 83913 ± 58325 | 39391 ± 31692 | 57500 ± 21850 | 0.001 | NS | NS |
| **Reduced Absorption at 280nm 0.1% (=1 g/l)** | 0.9779 ± 0.2736 | 0.2404 ± 0.4535 | 1.0472± 0.2294 | 0.001 | NS | 0.001 |
| **hydrogen (H)** | 0.50288 ± 0.00255 | 0.02504 ± 0.10891 | 0.48550± 0.00575 | 0.001 | NS | 0.001 |
| **Frequency of carbon (C)** | 0.31694 ±0.00328 | 0.01588 ± 0.06910 | 0.31325± 0.000931 | 0.001 | NS | 0.001 |
| **Frequency of nitrogen (N)** | 0.084313 ± 0.002626 | 0.00447± 0.01946 | 0.087500± 0.001592 | 0.001 | NS | 0.001 |
| **Frequency of oxygen (O)** | 0.093813± 0.003250 | 0.00487 ± 0.02120 | 0.11263± 0.00454 | 0.001 | NS | 0.001 |
| **Frequency of sulphur (S)** | 0.002000± 0.000516 | 0.000128 ±0.000588 | 0.001188± 0.000403 | 0.001 | 0.001 | 0.001 |
| **Frequency of Hydrophobic (A,F,G,I,L,M,P,V,W)** | 0.5063 ± 0.0524 | 0.02228 ± 0.09783 | 0.48250± 0.03272 | 0.001 | NS | 0.001 |
| **Frequency of Hydrophilic (C,N,Q,S,T,Y)** | 0.24881± 0.03243 | 0.01480 ± 0.06556 | 0.30025± 0.03162 | 0.001 | NS | 0.001 |
| **Frequency of Frequency of Other** | 0.2449 ± 0.0556 | 0.01331± 0.05831 | 0.21725± 0.03275 | 0.001 | NS | 0.001 |
| **Frequency of Negatively Charged (D & E)** | 0.11413 ±0.02771 | 0.00590 ± 0.02596 | 0.15100± 0.01953 | 0.001 | 0.001 | 0.001 |
| **Frequencyof Positively Charged (R & K)** | 0.10594± 0.03169 | 0.00596 ± 0.02628 | 0.05350± 0.02196 | 0.001 | 0.001 | 0.001 |
| **Frequency of Other** | 0.7798 ± 0.0529 | 0.0385 ± 0.1676 | 0.79525± 0.03028 | 0.001 | NS | 0.001 |
| **Freq of Alanine (A)** | 0.07100 ± 0.02088 | 0.003186 ± 0.015602 | 0.09913± 0.02325 | 0.001 | 0.001 | 0.001 |
| **Freq of Cysteine (C)** | 0.01075± 0.00458 | 0.000919 ± 0.004315 | 0.005250± 0.003376 | 0.001 | 0.001 | 0.001 |
| **Freq of Aspartic Acid (D)** | 0.04850 ± 0.01905 | 0.002574 ± 0.011450 | 0.09369± 0.01318 | 0.001 | 0.001 | 0.001 |
| **Freq of Glutamic Acid (E)** | 0.06563 ± 0.01862 | 0.003326± 0.014938 | 0.05781± 0.01388 | 0.001 | NS | 0.001 |
| **Freq of Phenylalanine (F)** | 0.04025 ± 0.01553 | 0.002151 ±0.009878 | 0.01619± 0.00404 | 0.001 | 0.001 | 0.001 |
| **Freq of Glycine (G)** | 0.07206 ± 0.01543 | 0.002884± 0.014025 | 0.11913 ± 0.03392 | 0.001 | 0.001 | 0.001 |
| **Freq of Histidine (H)** | 0.02463 ± 0.00742 | 0.001252 ± 0.005778 | 0.01288± 0.00443 | 0.001 | 0.001 | 0.001 |
| **Freq of Isoleucine (I)** | 0.05794 ± 0.00964 | 0.002578± 0.011698 | 0.03394± 0.00872 | 0.001 | 0.001 | 0.001 |
| **Freq of Lysine (K)** | 0.06213 ± 0.02763 | 0.003209 ± 0.014739 | 0.02169± 0.02337 | 0.001 | 0.001 | 0.001 |
| **Freq of Leucine (L)** | 0.11906 ± 0.01715 | 0.00462 ± 0.02074 | 0.06463± 0.01421 | 0.001 | 0.001 | 0.001 |
| **Freq of Methionine (M)** | 0.02063 ± 0.00678 | 0.001194± 0.005405 | 0.01200± 0.00994 | 0.001 | 0.001 | 0.001 |
| **Freq of Asparagine (N)** | 0.03531 ± 0.00971 | 0.002988± 0.014699 | 0.05375± 0.01624 | 0.001 | 0.001 | 0.001 |
| **Freq of Proline (P)** | 0.03963 ± 0.01362 | 0.002484 ± 0.011435 | 0.04138± 0.00419 | 0.001 | NS | 0.001 |
| **Freq of Glutamine (Q)** | 0.03244 ±0.00796 | 0.002039± 0.009535 | 0.03619± 0.00667 | 0.001 | NS | 0.001 |
| **Freq of Arginine (R)** | 0.04406 ± 0.00737 | 0.002756 ± 0.013030 | 0.03181± 0.00528 | 0.001 | 0.05 | 0.001 |
| **Freq of Serine (S)** | 0.08663± 0.02170 | 0.00485 ± 0.02213 | 0.09844± 0.02147 | 0.001 | NS | 0.001 |
| **Freq of Threonine (T)** | 0.05419 ± 0.01431 | 0.002589 ±0.011808 | 0.07169± 0.00593 | 0.001 | 0.001 | 0.001 |
| **Freq of Valine (V)** | 0.07356 ± 0.01192 | 0.002651 ± 0.012005 | 0.08450± 0.01145 | 0.001 | 0.05 | 0.001 |
| **Freq of Tryptophan (W)** | 0.01250 ± 0.00456 | 0.000523 ± 0.003215 | 0.011063± 0.003678 | 0.001 | NS | 0.001 |
| **Freq of Tyrosine (Y)** | 0.02938 ± 0.00717 | 0.001438 ± 0.006839 | 0.03500± 0.00731 | 0.001 | NS | 0.001 |
| **Percentage of Alanine (A)** | 7.094 ± 2.091 | 5.977 ± 2.789 | 9.918 ± 2.315 | NS | 0.01 | 0.001 |
| **Percentage of Cysteine (C)** | 1.064 ± 0.455 | 1.3201 ± 1.4773 | 0.5319 ± 0.3462 | NS | NS | NS |
| **Percentage of Aspartic Acid (D)** | 4.837 ± 1.886 | 5.521 ± 2.156 | 9.343 ± 1.314 | NS | 0.001 | 0.001 |
| **Percentage of Glutamic Acid (E)** | 6.574 ± 1.855 | 5.891 ± 2.588 | 5.757 ± 1.383 | NS | NS | NS |
| **Percentage of Phenylalanine (F)** | 4.006 ± 1.543 | 3.835 ± 2.309 | 1.6265 ± 0.3839 | NS | 0.01 | 0.001 |
| **Percentage of Glycine (G)** | 7.208 ± 1.546 | 6.018 ± 5.182 | 11.895 ± 3.409 | NS | 0.05 | 0.001 |
| **Percentage of Histidine (H)** | 2.475 ± 0.759 | 1.9503 ± 1.1032 | 1.290 ± 0.445 | NS | 0.05 | 0.05 |
| **Percentage of Isoleucine (I)** | 5.785 ± 0.960 | 5.134 ± 2.430 | 3.391 ± 0.863 | NS | 0.01 | 0.01 |
| **Percentage of Lysine (K)** | 6.209 ± 2.752 | 6.111 ± 2.302 | 2.174 ± 2.312 | NS | 0.001 | 0.001 |
| **Percentage of Leucine (L)** | 11.907 ± 1.714 | 8.931 ± 3.908 | 6.466 ± 1.423 | 0.01 | 0.001 | 0.05 |
| **Percentage of Methionine (M)** | 2.070 ± 0.673 | 1.7972 ± 1.1436 | 1.204 ± 1.003 | NS | NS | NS |
| **Percentage of Asparagine (N)** | 3.540 ± 0.965 | 4.604 ± 2.545 | 5.373 ± 1.610 | NS | NS | NS |
| **Percentage of Proline (P)** | 3.963 ± 1.353 | 4.497 ± 1.976 | 4.139 ± 0.429 | NS | NS | NS |
| **Percentage of Glutamine (Q)** | 3.253 ± 0.798 | 3.486 ± 2.043 | 3.629 ± 0.663 | NS | NS | NS |
| **Percentage of Arginine (R)** | 4.393 ± 0.740 | 4.303 ± 2.703 | 3.180 ± 0.526 | NS | NS | NS |
| **Percentage of Serine (S)** | 8.671 ± 2.172 | 8.372 ± 3.328 | 9.840 ± 2.134 | NS | NS | NS |
| **Percentage of Threonine (T)** | 5.424 ± 1.429 | 4.809 ± 1.808 | 7.166 ± 0.588 | NS | 0.05 | 0.001 |
| **Percentage of Valine (V)** | 7.349 ± 1.187 | 5.618 ± 2.107 | 8.461 ± 1.163 | 0.01 | NS | 0.001 |
| **Percentage of Tryptophan (W)** | 1.245 ± 0.463 | 0.4673 ± 0.6607 | 1.1186 ± 0.3876 | 0.001 | NS | 0.001 |
| **Percentage of Tyrosine (Y)** | 2.934 ±0.713 | 1.9309 ± 1.2951 | 3.498 ± 0.714 | 0.001 | NS | 0.001 |
| **Freq of Ala-Ala** | 0.006125 ± 0.003442 | 0.000357 ± 0.002558 | 0.01469 ± 0.00693 | 0.001 | 0.001 | 0.001 |
| **Freq of Ala-Cys** | 0.001375 ±0.001258 | 0.000109 ± 0.000686 | 0.001375 ± 0.001147 | 0.001 | NS | 0.001 |
| **Freq of Ala-Asp** | 0.002938 ± 0.002294 | 0.000128 ± 0.000893 | 0.011625 ± 0.003897 | 0.001 | 0.001 | 0.001 |
| **Freq of Ala-Glu** | 0.00550 ± 0.00501 | 0.000221 ± 0.001264 | 0.004625 ± 0.001996 | 0.001 | NS | 0.001 |
| **Freq of Ala-Phe** | 0.003938 ±0.003356 | 0.000143± 0.000997 | 0.001313 ± 0.001852 | 0.001 | 0.001 | 0.01 |
| **Freq of Ala-Gly** | 0.00400 ± 0.00434 | 0.000190 ± 0.001215 | 0.01213 ± 0.00535 | 0.001 | 0.001 | 0.001 |
| **Freq of Ala-His** | 0.001250 ±0.001291 | 0.0000388 ± 0.000276 | 0.001125 ± 0.001455 | 0.001 | NS | 0.001 |
| **Freq of Ala-Ile** | 0.002500± 0.002338 | 0.0000969 ± 0.000619 | 0.002875 ± 0.002029 | 0.001 | NS | 0.001 |
| **Freq of Ala-Lys** | 0.003563 ± 0.003577 | 0.000167 ± 0.000832 | 0.000375 ± 0.000719 | 0.001 | 0.001 | NS |
| **Freq of Ala-Leu** | 0.00913 ± 0.00459 | 0.000360 ± 0.002030 | 0.006313 ± 0.003219 | 0.001 | 0.001 | 0.01 |
| **Freq of Ala-Met** | 0.001438 ± 0.001548 | 0.0000814 ± 0.000709 | 0.000250 ± 0.000683 | 0.001 | 0.001 | NS |
| **Freq of Ala-Asn** | 0.002000 ± 0.001366 | 0.000132 ± 0.000850 | 0.003938 ± 0.003065 | 0.001 | 0.001 | 0.001 |
| **Freq of Ala-Pro** | 0.001875 ± 0.002125 | 0.000140± 0.000910 | 0.004625 ± 0.002754 | 0.001 | 0.001 | 0.001 |
| **Freq of Ala-Gln** | 0.002250 ± 0.002955 | 0.0000853± 0.000592 | 0.002000 ± 0.002309 | 0.001 | NS | 0.001 |
| **Freq of Ala-Arg** | 0.003063 ±0.001692 | 0.000186± 0.001068 | 0.002000 ± 0.001713 | 0.001 | 0.05 | 0.001 |
| **Freq of Ala-Ser** | 0.005313± 0.003459 | 0.000283± 0.001697 | 0.00663 ± 0.00563 | 0.001 | NS | 0.001 |
| **Freq of Ala-Thr** | 0.003188 ± 0.002834 | 0.000178 ± 0.001013 | 0.008625 ± 0.003722 | 0.001 | 0.001 | 0.001 |
| **Freq of Ala-Val** | 0.00869 ± 0.00411 | 0.000225 ± 0.001934 | 0.009125 ± 0.003557 | 0.001 | NS | 0.001 |
| **Freq of Ala-Trp** | 0.000187± 0.000403 | 0.0000271 ± 0.000323 | 0.002250 ± 0.000931 | NS | 0.001 | 0.001 |
| **Freq of Ala-Tyr** | 0.002813 ±0.001797 | 0.0000465 ± 0.000302 | 0.004000 ± 0.002366 | 0.001 | 0.001 | 0.001 |
| **Freq of Cys-Ala** | 0.000688 ± 0.000946 | 0.0000349 ± 0.000346 | 0.000375 ± 0.001025 | 0.001 | NS | 0.05 |
| **Freq of Cys-Cys** | 0.0000625±0.000250 | 0.00000775 ± 0.0000879 | 0.000000000±0.000000000 | NS | NS | NS |
| **Freq of Cys-Asp** | 0.001375 ± 0.001628 | 0.0000853 ± 0.000507 | 0.000125 ± 0.000342 | 0.001 | 0.001 | NS |
| **Freq of Cys-Glu** | 0.000875 ± 0.001628 | 0.0000814 ± 0.000797 | 0.001125 ± 0.001628 | 0.01 | NS | 0.001 |
| **Freq of Cys-Phe** | 0.000500 ± 0.000516 | 0.0000543 ± 0.000391 | 0.000000000±0.000000000 | 0.001 | 0.001 | NS |
| **Freq of Cys-Gly** | 0.000375 ± 0.001025 | 0.0000659 ± 0.000571 | 0.000500 ± 0.000894 | NS | NS | 0.05 |
| **Freq of Cys-His** | 0.000625 ± 0.001708 | 0.0000853 ± 0.000649 | 0.000125 ± 0.000500 | 0.05 | NS | NS |
| **Freq of Cys-Ile** | 0.000563 ± 0.001031 | 0.0000465 ± 0.000371 | 0.000000000±0.000000000 | 0.001 | 0.001 | NS |
| **Freq of Cys-Lys** | 0.000187 ± 0.000403 | 0.0000233 ± 0.000196 | 0.000000000±0.000000000 | 0.01 | 0.05 | NS |
| **Freq of Cys-Leu** | 0.001250 ± 0.001571 | 0.0000659 ± 0.000441 | 0.000000000±0.000000000 | 0.001 | 0.001 | NS |
| **Freq of Cys-Met** | 0.000188 ± 0.000403 | 0.00000775 ± 0.0000879 | 0.000000000±0.000000000 | 0.001 | 0.001 | NS |
| **Freq of Cys-Asn** | 0.000188 ± 0.000750 | 0.0000116 ± 0.000139 | 0.000000000±0.000000000 | 0.01 | 0.05 | NS |
| **Freq of Cys-Pro** | 0.000313 ± 0.000793 | 0.0000271 ± 0.000436 | 0.000750 ± 0.000931 | NS | 0.05 | 0.001 |
| **Freq of Cys-Gln** | 0.000000000±0.000000000 | 0.00000775 ± 0.0000879 | 0.000125 ± 0.000500 | NS | 0.05 | 0.01 |
| **Freq of Cys-Arg** | 0.001250 ± 0.001653 | 0.0000853 ± 0.000846 | 0.000000000±0.000000000 | 0.001 | 0.001 | NS |
| **Freq of Cys-Ser** | 0.000750 ± 0.001528 | 0.000101 ± 0.000687 | 0.001125 ± 0.001628 | 0.01 | NS | 0.001 |
| **Freq of Cys-Thr** | 0.000125 ± 0.000342 | 0.0000388 ± 0.000246 | 0.000125 ± 0.000342 | NS | NS | NS |
| **Freq of Cys-Val** | 0.000375 ± 0.000806 | 0.0000465 ± 0.000456 | 0.000500 ± 0.001155 | 0.05 | NS | 0.01 |
| **Freq of Cys-Trp** | 0.000188 ± 0.000544 | 0.0000310 ± 0.000383 | 0.000625 ± 0.000957 | NS | 0.05 | 0.001 |
| **Freq of Cys-Tyr** | 0.000875 ± 0.000957 | 0.0000116 ± 0.000139 | 0.000000000±0.000000000 | 0.001 | 0.001 | NS |
| **Freq of Asp-Ala** | 0.003188 ± 0.003291 | 0.000128± 0.000901 | 0.007187 ± 0.003920 | 0.001 | 0.001 | 0.001 |
| **Freq of Asp-Cys** | 0.000563 ± 0.001632 | 0.0000155 ± 0.000152 | 0.000250 ± 0.000683 | 0.001 | NS | NS |
| **Freq of Asp-Asp** | 0.003188 ± 0.002857 | 0.000116 ± 0.000922 | 0.00850 ± 0.00750 | 0.001 | 0.001 | 0.001 |
| **Freq of Asp-Glu** | 0.003750 ± 0.002720 | 0.000310 ± 0.001673 | 0.00581 ± 0.00402 | 0.001 | NS | 0.001 |
| **Freq of Asp-Phe** | 0.002000 ± 0.002066 | 0.0000969 ±0.000546 | 0.003938 ± 0.000772 | 0.001 | 0.001 | 0.001 |
| **Freq of Asp-Gly** | 0.004125 ± 0.001996 | 0.000178 ± 0.000916 | 0.01206 ± 0.00892 | 0.001 | 0.001 | 0.001 |
| **Freq of Asp-His** | 0.000938 ± 0.001237 | 0.0000194 ±0.000138 | 0.001813 ± 0.002073 | 0.001 | 0.001 | 0.001 |
| **Freq of Asp-Ile** | 0.003438 ± 0.003483 | 0.0000853 ±0.000630 | 0.003188 ± 0.002007 | 0.001 | NS | 0.001 |
| **Freq of Asp-Lys** | 0.002063 ± 0.002048 | 0.000116 ± 0.000707 | 0.000500 ± 0.000816 | 0.001 | 0.001 | NS |
| **Freq of Asp-Leu** | 0.004750 ± 0.003838 | 0.000306 ±0.001599 | 0.007188 ± 0.002689 | 0.001 | 0.001 | 0.001 |
| **Freq of Asp-Met** | 0.001375 ± 0.001668 | 0.0000465 ± 0.000361 | 0.000625 ± 0.001408 | 0.001 | 0.001 | 0.001 |
| **Freq of Asp-Asn** | 0.001500 ± 0.001366 | 0.000159 ± 0.000865 | 0.004250 ± 0.002017 | 0.001 | 0.001 | 0.001 |
| **Freq of Asp-Pro** | 0.003250 ± 0.002463 | 0.0000736 ± 0.000482 | 0.009500 ± 0.001966 | 0.001 | 0.001 | 0.001 |
| **Freq of Asp-Gln** | 0.001125 ± 0.002277 | 0.000120 ± 0.000928 | 0.004750 ± 0.001612 | 0.001 | 0.001 | 0.001 |
| **Freq of Asp-Arg** | 0.001625 ± 0.002306 | 0.000225± 0.001559 | 0.003125 ± 0.003160 | 0.01 | 0.05 | 0.001 |
| **Freq of Asp-Ser** | 0.004313 ± 0.003535 | 0.000217 ± 0.001098 | 0.006750 ± 0.003890 | 0.001 | 0.001 | 0.001 |
| **Freq of Asp-Thr** | 0.001688 ± 0.001778 | 0.000112 ± 0.000688 | 0.006750 ± 0.002463 | 0.001 | 0.001 | 0.001 |
| **Freq of Asp-Val** | 0.002500 ± 0.002309 | 0.000171 ± 0.001030 | 0.005625 ± 0.003160 | 0.001 | 0.001 | 0.001 |
| **Freq of Asp-Trp** | 0.002063 ± 0.002205 | 0.0000116± 0.000187 | 0.000125 ± 0.000500 | 0.001 | 0.001 | NS |
| **Freq of Asp-Tyr** | 0.001750 ± 0.001844 | 0.0000930 ± 0.000716 | 0.002750 ± 0.002380 | 0.001 | 0.01 | 0.001 |
| **Freq of Glu-Ala** | 0.004938 ± 0.002863 | 0.000248 ± 0.001487 | 0.003688 ± 0.001740 | 0.001 | NS | 0.001 |
| **Freq of Glu-Cys** | 0.000187 ±0.000750 | 0.0000504 ± 0.000425 | 0.000500 ± 0.000894 | NS | NS | 0.001 |
| **Freq of Glu-Asp** | 0.003250 ± 0.002517 | 0.000217± 0.001109 | 0.002938 ± 0.001879 | 0.001 | NS | 0.001 |
| **Freq of Glu-Glu** | 0.00769 ± 0.00623 | 0.000295 ± 0.002223 | 0.00569 ± 0.00520 | 0.001 | NS | 0.001 |
| **Freq of Glu-Phe** | 0.001500 ±0.003464 | 0.000163 ± 0.001061 | 0.000875 ± 0.001025 | 0.001 | NS | NS |
| **Freq of Glu-Gly** | 0.00706 ± 0.00678 | 0.000140± 0.000948 | 0.004000 ± 0.002898 | 0.001 | 0.001 | 0.001 |
| **Freq of Glu-His** | 0.000938 ± 0.001181 | 0.0000736 ± 0.000542 | 0.000500 ± 0.001033 | 0.001 | NS | 0.05 |
| **Freq of Glu-Ile** | 0.003500± 0.002098 | 0.000209 ±0.001211 | 0.004375 ± 0.002895 | 0.001 | NS | 0.001 |
| **Freq of Glu-Lys** | 0.004625 ± 0.003364 | 0.000256± 0.001363 | 0.001250 ± 0.002817 | 0.001 | 0.001 | 0.05 |
| **Freq of Glu-Leu** | 0.00638 ±0.00492 | 0.000372 ± 0.001884 | 0.007188 ± 0.002857 | 0.001 | NS | 0.001 |
| **Freq of Glu-Met** | 0.002500 ±0.002221 | 0.0000194 ± 0.000164 | 0.000000000±0.000000000 | 0.001 | 0.001 | NS |
| **Freq of Glu-Asn** | 0.002375 ±0.002029 | 0.000225 ± 0.001252 | 0.002125 ± 0.002363 | 0.001 | NS | 0.001 |
| **Freq of Glu-Pro** | 0.001500 ±0.001633 | 0.000143 ± 0.001017 | 0.00275 ± 0.00412 | 0.001 | 0.05 | 0.001 |
| **Freq of Glu-Gln** | 0.001250± 0.001807 | 0.000101± 0.000640 | 0.002813 ± 0.002588 | 0.001 | 0.001 | 0.001 |
| **Freq of Glu-Arg** | 0.002063 ± 0.001652 | 0.000147 ± 0.000861 | 0.002438 ± 0.001825 | 0.001 | NS | 0.001 |
| **Freq of Glu-Ser** | 0.007000 ±0.003651 | 0.000233± 0.001248 | 0.00613 ± 0.00503 | 0.001 | NS | 0.001 |
| **Freq of Glu-Thr** | 0.002813 ± 0.002198 | 0.0000969 ± 0.000619 | 0.004375 ± 0.003344 | 0.001 | 0.001 | 0.001 |
| **Freq of Glu-Val** | 0.003250 ± 0.002408 | 0.000190± 0.001119 | 0.002250 ± 0.002266 | 0.001 | NS | 0.001 |
| **Freq of Glu-Trp** | 0.000875 ± 0.001360 | 0.0000581 ± 0.000643 | 0.000125 ± 0.000500 | 0.001 | 0.01 | NS |
| **Freq of Glu-Tyr** | 0.002125 ±0.003594 | 0.0000930± 0.000604 | 0.004000 ± 0.001366 | 0.001 | 0.001 | 0.001 |
| **Freq of Phe-Ala** | 0.001813± 0.002786 | 0.0000969 ± 0.000567 | 0.000186 ± 0.000919 | 0.001 | 0.001 | NS |
| **Freq of Phe-Cys** | 0.000875± 0.000885 | 0.0000504 ±0.000485 | 0.0000931 ± 0.000534 | 0.001 | 0.001 | NS |
| **Freq of Phe-Asp** | 0.001125 ± 0.002277 | 0.000101 ± 0.000876 | 0.000266 ± 0.001132 | 0.001 | 0.05 | 0.001 |
| **Freq of Phe-Glu** | 0.002500± 0.001592 | 0.000109 ± 0.000633 | 0.000255 ± 0.000906 | 0.001 | 0.001 | NS |
| **Freq of Phe-Phe** | 0.001438 ±0.001413 | 0.0000736 ± 0.000520 | 0.000172 ± 0.000738 | 0.001 | 0.001 | 0.05 |
| **Freq of Phe-Gly** | 0.003125 ±0.002094 | 0.000128± 0.000960 | 0.000410± 0.001377 | 0.001 | NS | 0.001 |
| **Freq of Phe-His** | 0.001000 ±0.001713 | 0.0000465± 0.000371 | 0.0000966± 0.000568 | 0.001 | 0.001 | NS |
| **Freq of Phe-Ile** | 0.002938 ±0.001914 | 0.000124 ± 0.000895 | 0.000286± 0.001158 | 0.001 | 0.001 | NS |
| **Freq of Phe-Lys** | 0.002125 ± 0.002655 | 0.0000814± 0.000534 | 0.000245± 0.000991 | 0.001 | 0.001 | 0.001 |
| **Freq of Phe-Leu** | 0.00644 ±0.00538 | 0.000167± 0.001084 | 0.000579± 0.002168 | 0.001 | 0.001 | 0.05 |
| **Freq of Phe-Met** | 0.000313 ± 0.000793 | 0.0000736 ± 0.000773 | 0.000117 ± 0.000824 | NS | NS | 0.05 |
| **Freq of Phe-Asn** | 0.001500 ± 0.001265 | 0.000120 ± 0.000787 | 0.000245 ± 0.001074 | 0.001 | NS | 0.01 |
| **Freq of Phe-Pro** | 0.002875± 0.001544 | 0.0000543 ± 0.000370 | 0.000255± 0.000855 | 0.001 | 0.001 | 0.001 |
| **Freq of Phe-Gln** | 0.000750 ± 0.001238 | 0.000143 ±0.000798 | 0.000169± 0.000817 | 0.01 | 0.05 | NS |
| **Freq of Phe-Arg** | 0.001063 ±0.001340 | 0.000124 ±0.000929 | 0.000197± 0.000977 | 0.001 | NS | NS |
| **Freq of Phe-Ser** | 0.002063 ±0.002112 | 0.000291± 0.001504 | 0.000524 ± 0.001719 | 0.001 | NS | 0.001 |
| **Freq of Phe-Thr** | 0.002250 ±0.001880 | 0.0000891 ± 0.000568 | 0.000241± 0.000910 | 0.001 | 0.001 | 0.05 |
| **Freq of Phe-Val** | 0.00431 ±0.00490 | 0.000159 ± 0.001063 | 0.000490 ± 0.001840 | 0.001 | 0.001 | 0.001 |
| **Freq of Phe-Trp** | 0.000875 ± 0.000957 | 0.0000543 ±0.000496 | 0.0000966 ± 0.000550 | 0.001 | 0.001 | NS |
| **Freq of Phe-Tyr** | 0.001125 ±0.001258 | 0.0000620± 0.000463 | 0.000117± 0.000576 | 0.001 | 0.001 | NS |
| **Freq of Gly-Ala** | 0.00725 ± 0.00488 | 0.000225 ± 0.001399 | 0.000983 ± 0.002922 | 0.001 | NS | 0.001 |
| **Freq of Gly-Cys** | 0.000875 ± 0.001962 | 0.0000194 ± 0.000206 | 0.0000793 ± 0.000549 | 0.001 | 0.01 | NS |
| **Freq of Gly-Asp** | 0.003563 ± 0.003705 | 0.000236 ± 0.001365 | 0.001131± 0.004000 | 0.001 | 0.001 | 0.001 |
| **Freq of Gly-Glu** | 0.004438 ± 0.003483 | 0.000140± 0.000835 | 0.000531 ± 0.001693 | 0.001 | 0.01 | 0.001 |
| **Freq of Gly-Phe** | 0.002125 ± 0.001784 | 0.000128 ± 0.000996 | 0.000334 ± 0.001251 | 0.001 | NS | 0.001 |
| **Freq of Gly-Gly** | 0.004563 ± 0.003502 | 0.000326± 0.002660 | 0.001790± 0.006565 | 0.001 | 0.001 | 0.001 |
| **Freq of Gly-His** | 0.000688 ± 0.001138 | 0.0000930 ±0.000933 | 0.000252± 0.001229 | NS | 0.001 | 0.001 |
| **Freq of Gly-Ile** | 0.00675 ± 0.00401 | 0.000217± 0.001143 | 0.000862± 0.002378 | 0.001 | 0.05 | 0.001 |
| **Freq of Gly-Lys** | 0.003313 ± 0.002152 | 0.000205 ± 0.001190 | 0.000407 ± 0.001434 | 0.001 | 0.001 | NS |
| **Freq of Gly-Leu** | 0.00844 ± 0.00612 | 0.000136± 0.000739 | 0.000934± 0.002816 | 0.001 | 0.001 | 0.001 |
| **Freq of Gly-Met** | 0.001500 ± 0.001713 | 0.0000465 ± 0.000456 | 0.000138 ± 0.000682 | 0.001 | 0.001 | NS |
| **Freq of Gly-Asn** | 0.001937 ± 0.001731 | 0.000182 ±0.001191 | 0.000690 ± 0.002199 | 0.001 | 0.001 | 0.001 |
| **Freq of Gly-Pro** | 0.001563 ± 0.001263 | 0.000132± 0.000965 | 0.000421± 0.001393 | 0.001 | 0.001 | 0.001 |
| **Freq of Gly-Gln** | 0.002625 ± 0.002553 | 0.000136 ± 0.000955 | 0.000362± 0.001309 | 0.001 | NS | 0.001 |
| **Freq of Gly-Arg** | 0.002500 ± 0.002221 | 0.0000775± 0.000580 | 0.000579 ± 0.001930 | 0.001 | 0.001 | 0.001 |
| **Freq of Gly-Ser** | 0.00725 ± 0.00452 | 0.000252 ±0.001324 | 0.001359 ± 0.003856 | 0.001 | 0.001 | 0.001 |
| **Freq of Gly-Thr** | 0.003750 ± 0.002955 | 0.000132 ±0.000773 | 0.000766 ± 0.002389 | 0.001 | 0.001 | 0.001 |
| **Freq of Gly-Val** | 0.005938 ± 0.003454 | 0.000174± 0.001208 | 0.001117 ± 0.003318 | 0.001 | 0.001 | 0.001 |
| **Freq of Gly-Trp** | 0.000250 ± 0.000775 | 0.0000116 ± 0.000187 | 0.0000517 ± 0.000344 | 0.05 | NS | 0.001 |
| **Freq of Gly-Tyr** | 0.003313 ± 0.003842 | 0.0000698 ± 0.000546 | 0.000431± 0.001475 | 0.001 | NS | 0.001 |
| **Freq of His-Ala** | 0.001000 ± 0.001211 | 0.0000465 ± 0.000338 | 0.000148 ± 0.000630 | 0.001 | NS | 0.001 |
| **Freq of His-Cys** | 0.0000625 ± 0.000250 | 0.0000504 ± 0.000396 | 0.0000483 ± 0.000378 | NS | NS | NS |
| **Freq of His-Asp** | 0.001563 ± 0.001711 | 0.0000426 ± 0.000397 | 0.000148 ± 0.000712 | 0.001 | 0.001 | 0.05 |
| **Freq of His-Glu** | 0.002125 ± 0.002705 | 0.0000659 ± 0.000514 | 0.000252 ± 0.001020 | 0.001 | 0.05 | 0.001 |
| **Freq of His-Phe** | 0.001438 ± 0.001315 | 0.0000814 ± 0.000583 | 0.000152 ± 0.000699 | 0.001 | 0.001 | NS |
| **Freq of His-Gly** | 0.002000 ± 0.001897 | 0.0000349 ± 0.000297 | 0.000293 ± 0.000941 | 0.001 | 0.001 | 0.001 |
| **Freq of His-His** | 0.000688 ± 0.000704 | 0.0000504 ± 0.000580 | 0.0000828 ± 0.000588 | 0.001 | 0.01 | NS |
| **Freq of His-Ile** | 0.000375 ± 0.000806 | 0.0000426 ± 0.000377 | 0.0000862 ± 0.000522 | 0.05 | NS | 0.01 |
| **Freq of His-Lys** | 0.000563 ± 0.001413 | 0.000105 ± 0.000921 | 0.000124 ± 0.000933 | NS | NS | NS |
| **Freq of His-Leu** | 0.002313 ± 0.002152 | 0.0000930 ± 0.000584 | 0.000272 ± 0.000948 | 0.001 | 0.001 | 0.001 |
| **Freq of His-Met** | 0.000000000±0.000000000 | 0.0000116 ± 0.000107 | 0.0000103 ± 0.000101 | NS | NS | NS |
| **Freq of His-Asn** | 0.001250 ± 0.001732 | 0.0000620 ± 0.000518 | 0.000131 ± 0.000694 | 0.001 | 0.001 | NS |
| **Freq of His-Pro** | 0.001813 ± 0.001682 | 0.0000581 ± 0.000424 | 0.000241 ± 0.000778 | 0.001 | NS | 0.001 |
| **Freq of His-Gln** | 0.002313 ± 0.001815 | 0.0000891 ± 0.000595 | 0.000207 ± 0.000864 | 0.001 | 0.001 | NS |
| **Freq of His-Arg** | 0.001500 ± 0.001592 | 0.000143 ± 0.001366 | 0.000231 ± 0.001384 | 0.001 | 0.05 | NS |
| **Freq of His-Ser** | 0.002188 ± 0.002105 | 0.0000853 ± 0.000544 | 0.000217 ± 0.000871 | 0.001 | 0.001 | NS |
| **Freq of His-Thr** | 0.002438 ± 0.003705 | 0.0000698 ± 0.000510 | 0.000266 ± 0.001180 | 0.001 | 0.01 | 0.001 |
| **Freq of His-Val** | 0.001250 ± 0.001880 | 0.0000853 ± 0.000666 | 0.000262 ± 0.000970 | 0.001 | 0.01 | 0.001 |
| **Freq of His-Trp** | 0.000000000±0.000000000 | 0.000000000±0.000000000 | 0.000000000±0.000000000 | 0 | 0 | 0 |
| **Freq of His-Tyr** | 0.000313 ± 0.000479 | 0.0000310 ± 0.000317 | 0.0000448 ± 0.000324 | 0.01 | 0.05 | NS |
| **Freq of Ile-Ala** | 0.005375 ± 0.003649 | 0.000147 ± 0.000934 | 0.000679 ± 0.002010 | 0.001 | NS | 0.001 |
| **Freq of Ile-Cys** | 0.000813 ± 0.001223 | 0.0000698 ± 0.000517 | 0.000107 ± 0.000587 | 0.001 | 0.001 | NS |
| **Freq of Ile-Asp** | 0.003750 ± 0.003661 | 0.000140 ± 0.000902 | 0.000555 ± 0.001801 | 0.001 | NS | 0.001 |
| **Freq of Ile-Glu** | 0.003188 ±0.003544 | 0.000163 ± 0.000858 | 0.000476 ± 0.001502 | 0.001 | NS | 0.001 |
| **Freq of Ile-Phe** | 0.002563± 0.002394 | 0.000120 ± 0.000945 | 0.000248 ± 0.001185 | 0.001 | 0.001 | NS |
| **Freq of Ile-Gly** | 0.004750 ± 0.002817 | 0.000147 ± 0.001018 | 0.000597 ± 0.001803 | 0.001 | 0.05 | 0.001 |
| **Freq of Ile-His** | 0.001688 ± 0.002522 | 0.0000775 ± 0.000508 | 0.000190 ± 0.000862 | 0.001 | 0.001 | NS |
| **Freq of Ile-Ile** | 0.003000± 0.002129 | 0.000116 ± 0.000713 | 0.000338 ± 0.001139 | 0.001 | 0.001 | 0.001 |
| **Freq of Ile-Lys** | 0.003000± 0.001826 | 0.000190 ± 0.001133 | 0.000362 ± 0.001330 | 0.001 | 0.001 | NS |
| **Freq of Ile-Leu** | 0.004438± 0.003829 | 0.000202 ± 0.001153 | 0.000559 ± 0.001860 | 0.001 | 0.001 | 0.001 |
| **Freq of Ile-Met** | 0.000750± 0.001183 | 0.0000388 ± 0.000262 | 0.000103 ± 0.000516 | 0.001 | NS | 0.001 |
| **Freq of Ile-Asn** | 0.00425 ± 0.00404 | 0.000140 ± 0.000816 | 0.000448 ± 0.001558 | 0.001 | 0.001 | 0.001 |
| **Freq of Ile-Pro** | 0.001813 ± 0.001642 | 0.000155 ± 0.000886 | 0.000272 ± 0.001018 | 0.001 | 0.001 | NS |
| **Freq of Ile-Gln** | 0.001625 ± 0.002125 | 0.0000736 ± 0.000421 | 0.000217 ± 0.000851 | 0.001 | NS | 0.001 |
| **Freq of Ile-Arg** | 0.002875 ±0.002062 | 0.000136 ± 0.000795 | 0.000279 ± 0.001085 | 0.001 | 0.001 | NS |
| **Freq of Ile-Ser** | 0.004063± 0.002645 | 0.000267 ± 0.001448 | 0.000745 ± 0.002225 | 0.001 | NS | 0.001 |
| **Freq of Ile-Thr** | 0.003375 ±0.002941 | 0.000143 ± 0.000808 | 0.000383 ± 0.001345 | 0.001 | 0.001 | 0.001 |
| **Freq of Ile-Val** | 0.00425 ± 0.00451 | 0.000109 ± 0.000657 | 0.000524 ± 0.001707 | 0.001 | NS | 0.001 |
| **Freq of Ile-Trp** | 0.001438 ± 0.002250 | 0.0000116 ± 0.000139 | 0.0000897 ± 0.000622 | 0.001 | 0.001 | NS |
| **Freq of Ile-Tyr** | 0.001125 ±0.001586 | 0.000151 ± 0.001004 | 0.000245 ± 0.001109 | 0.001 | NS | 0.05 |
| **Freq of Lys-Ala** | 0.003625 ± 0.001310 | 0.000213 ± 0.001076 | 0.000562 ± 0.001506 | 0.001 | NS | 0.001 |
| **Freq of Lys-Cys** | 0.0000625± 0.000250 | 0.000112 ± 0.000694 | 0.000103 ± 0.000657 | NS | NS | NS |
| **Freq of Lys-Asp** | 0.002625± 0.003030 | 0.000116 ± 0.000734 | 0.000372 ± 0.001359 | 0.001 | NS | 0.001 |
| **Freq of Lys-Glu** | 0.00719 ± 0.00625 | 0.000267 ±0.001738 | 0.000676 ± 0.002711 | 0.001 | 0.001 | NS |
| **Freq of Lys-Phe** | 0.002563 ±0.001825 | 0.000159 ± 0.001251 | 0.000324 ± 0.001409 | 0.001 | 0.001 | NS |
| **Freq of Lys-Gly** | 0.00300 ±0.00408 | 0.0000930 ± 0.000591 | 0.000290 ± 0.001315 | 0.001 | 0.001 | NS |
| **Freq of Lys-His** | 0.001500 ± 0.001633 | 0.0000426 ± 0.000356 | 0.000121 ± 0.000602 | 0.001 | 0.001 | NS |
| **Freq of Lys-Ile** | 0.003563 ±0.001896 | 0.000190 ±0.001091 | 0.000434 ± 0.001408 | 0.001 | 0.001 | 0.001 |
| **Freq of Lys-Lys** | 0.00563 ±0.00624 | 0.000295 ± 0.001583 | 0.000697 ± 0.002581 | 0.001 | 0.001 | 0.01 |
| **Freq of Lys-Leu** | 0.00863 ± 0.00578 | 0.000283 ± 0.001415 | 0.000790 ± 0.002694 | 0.001 | 0.001 | NS |
| **Freq of Lys-Met** | 0.001125 ±0.001628 | 0.0000775 ± 0.000685 | 0.000131 ± 0.000783 | 0.001 | 0.001 | NS |
| **Freq of Lys-Asn** | 0.00388 ± 0.00446 | 0.000194± 0.001141 | 0.000434± 0.001760 | 0.001 | 0.001 | NS |
| **Freq of Lys-Pro** | 0.001938 ±0.001652 | 0.000229 ± 0.001293 | 0.000352 ± 0.001377 | 0.001 | 0.05 | NS |
| **Freq of Lys-Gln** | 0.00300 ± 0.00523 | 0.000163 ± 0.000898 | 0.000434 ± 0.001891 | 0.001 | NS | 0.001 |
| **Freq of Lys-Arg** | 0.002438 ±0.001999 | 0.000248 ± 0.001318 | 0.000459 ± 0.001513 | 0.001 | NS | 0.001 |
| **Freq of Lys-Ser** | 0.003625 ± 0.002941 | 0.000248 ± 0.001395 | 0.000441± 0.001682 | 0.001 | 0.001 | NS |
| **Freq of Lys-Thr** | 0.003000 ± 0.003183 | 0.000105 ± 0.000712 | 0.000259 ± 0.001191 | 0.001 | 0.001 | NS |
| **Freq of Lys-Val** | 0.003250± 0.002620 | 0.0000930± 0.000642 | 0.000317 ± 0.001287 | 0.001 | 0.001 | 0.01 |
| **Freq of Lys-Trp** | 0.000438 ± 0.000512 | 0.0000543 ± 0.000438 | 0.0000724 ± 0.000438 | 0.01 | 0.05 | NS |
| **Freq of Lys-Tyr** | 0.001313± 0.001302 | 0.0000698 ± 0.000436 | 0.000224 ± 0.000824 | 0.001 | NS | 0.001 |
| **Freq of Leu-Ala** | 0.007313 ±0.003198 | 0.000322± 0.002090 | 0.001031 ± 0.003014 | 0.001 | NS | 0.001 |
| **Freq of Leu-Cys** | 0.002750 ±0.003435 | 0.0000891 ± 0.000719 | 0.000231 ± 0.001202 | 0.001 | 0.001 | NS |
| **Freq of Leu-Asp** | 0.00544 ± 0.00479 | 0.000291 ± 0.001408 | 0.000824 ± 0.002375 | 0.001 | NS | 0.001 |
| **Freq of Leu-Glu** | 0.00738± 0.00449 | 0.000302 ±0.001477 | 0.000824 ± 0.002438 | 0.001 | 0.001 | 0.001 |
| **Freq of Leu-Phe** | 0.004000 ± 0.003286 | 0.000260 ± 0.001357 | 0.000472 ± 0.001721 | 0.001 | 0.001 | NS |
| **Freq of Leu-Gly** | 0.01075 ± 0.00451 | 0.000225 ± 0.001286 | 0.001334 ± 0.003799 | 0.001 | NS | 0.001 |
| **Freq of Leu-His** | 0.003813 ± 0.001797 | 0.000128 ± 0.000796 | 0.000331 ± 0.001206 | 0.001 | 0.001 | NS |
| **Freq of Leu-Ile** | 0.007500± 0.003899 | 0.000217 ± 0.001213 | 0.000672 ± 0.002243 | 0.001 | 0.001 | 0.05 |
| **Freq of Leu-Lys** | 0.006688 ± 0.003478 | 0.000287 ± 0.001596 | 0.000769 ± 0.002342 | 0.001 | 0.001 | 0.001 |
| **Freq of Leu-Leu** | 0.01669 ± 0.00662 | 0.000434 ± 0.002337 | 0.001507 ± 0.004696 | 0.001 | 0.001 | 0.001 |
| **Freq of Leu-Met** | 0.002000 ± 0.001633 | 0.000143 ± 0.000813 | 0.000238 ± 0.000953 | 0.001 | 0.001 | NS |
| **Freq of Leu-Asn** | 0.003625± 0.001408 | 0.000333 ± 0.001675 | 0.000669 ±0.002116 | 0.001 | NS | 0.001 |
| **Freq of Leu-Pro** | 0.005250 ±0.002436 | 0.000329 ± 0.001601 | 0.000728 ± 0.002052 | 0.001 | 0.001 | 0.001 |
| **Freq of Leu-Gln** | 0.002688 ± 0.001815 | 0.000147± 0.000833 | 0.000390 ± 0.001175 | 0.001 | NS | 0.001 |
| **Freq of Leu-Arg** | 0.006688 ± 0.002120 | 0.000233 ± 0.001232 | 0.000755 ± 0.002094 | 0.001 | 0.001 | 0.001 |
| **Freq of Leu-Ser** | 0.01100 ± 0.00505 | 0.000403 ± 0.001971 | 0.001555 ± 0.004158 | 0.001 | NS | 0.001 |
| **Freq of Leu-Thr** | 0.004750 ± 0.003751 | 0.000233 ± 0.001229 | 0.000752 ± 0.002103 | 0.001 | NS | 0.001 |
| **Freq of Leu-Val** | 0.006125 ±0.002553 | 0.000171± 0.001088 | 0.000769 ± 0.002109 | 0.001 | 0.05 | 0.001 |
| **Freq of Leu-Trp** | 0.001438 ± 0.001459 | 0.0000271 ± 0.000185 | 0.000110 ± 0.000508 | 0.001 | 0.001 | NS |
| **Freq of Leu-Tyr** | 0.003250± 0.002295 | 0.000120 ± 0.000747 | 0.000362 ± 0.001190 | 0.001 | 0.001 | 0.001 |
| **Freq of Met-Ala** | 0.001063 ± 0.001124 | 0.000101 ± 0.000915 | 0.000334± 0.001223 | 0.001 | 0.001 | 0.001 |
| **Freq of Met-Cys** | 0.000187 ± 0.000403 | 0.00000388± 0.0000623 | 0.0000138 ± 0.000117 | 0.001 | 0.001 | NS |
| **Freq of Met-Asp** | 0.000750± 0.001000 | 0.0000620 ± 0.000399 | 0.000152± 0.000609 | 0.001 | NS | 0.001 |
| **Freq of Met-Glu** | 0.001500 ± 0.001592 | 0.0000659 ± 0.000449 | 0.000162± 0.000689 | 0.001 | 0.001 | NS |
| **Freq of Met-Phe** | 0.000688± 0.000793 | 0.0000504 ± 0.000415 | 0.000110 ± 0.000560 | 0.001 | NS | 0.01 |
| **Freq of Met-Gly** | 0.001625 ± 0.001544 | 0.0000349 ± 0.000297 | 0.000148 ± 0.000662 | 0.001 | 0.001 | 0.01 |
| **Freq of Met-His** | 0.000438 ± 0.000814 | 0.0000271 ± 0.000205 | 0.0000483 ± 0.000284 | 0.001 | 0.001 | NS |
| **Freq of Met-Ile** | 0.001250 ±0.001571 | 0.0000775 ± 0.000545 | 0.000159± 0.000708 | 0.001 | 0.001 | NS |
| **Freq of Met-Lys** | 0.000375± 0.000500 | 0.0000543 ± 0.000381 | 0.000117 ± 0.000539 | 0.05 | 0.05 | 0.001 |
| **Freq of Met-Leu** | 0.002438 ± 0.001711 | 0.000109 ± 0.000595 | 0.000238 ± 0.000874 | 0.001 | 0.001 | NS |
| **Freq of Met-Met** | 0.0000625 ± 0.000250 | 0.0000233 ± 0.000175 | 0.0000241 ± 0.000175 | NS | NS | NS |
| **Freq of Met-Asn** | 0.001500 ± 0.001633 | 0.000120 ± 0.000854 | 0.000190 ± 0.000942 | 0.001 | 0.001 | NS |
| **Freq of Met-Pro** | 0.000438 ± 0.000814 | 0.0000698 ± 0.000614 | 0.000114 ± 0.000694 | NS | NS | 0.05 |
| **Freq of Met-Gln** | 0.000938 ± 0.001237 | 0.0000271 ± 0.000223 | 0.000103 ± 0.000467 | 0.001 | 0.01 | 0.001 |
| **Freq of Met-Arg** | 0.002125± 0.001857 | 0.0000659 ± 0.000514 | 0.000183± 0.000801 | 0.001 | 0.001 | NS |
| **Freq of Met-Ser** | 0.001188 ±0.001601 | 0.000124± 0.000764 | 0.000290 ± 0.001035 | 0.001 | 0.05 | 0.001 |
| **Freq of Met-Thr** | 0.001625 ± 0.001784 | 0.0000891 ± 0.000651 | 0.000197 ± 0.000840 | 0.001 | 0.001 | NS |
| **Freq of Met-Val** | 0.002375 ± 0.002446 | 0.0000543 ± 0.000464 | 0.000241 ± 0.001099 | 0.001 | 0.001 | 0.001 |
| **Freq of Met-Trp** | 0.000188± 0.000750 | 0.0000271 ± 0.000323 | 0.0000345 ± 0.000351 | NS | NS | NS |
| **Freq of Met-Tyr** | 0.000688 ± 0.001702 | 0.0000194 ± 0.000206 | 0.0000621 ± 0.000474 | 0.001 | 0.001 | NS |
| **Freq of Asn-Ala** | 0.001688 ± 0.002301 | 0.000155 ± 0.000933 | 0.000638 ± 0.002079 | 0.001 | 0.001 | 0.001 |
| **Freq of Asn-Cys** | 0.000187 ± 0.000750 | 0.0000659 ± 0.000413 | 0.000159 ± 0.000673 | NS | 0.001 | 0.001 |
| **Freq of Asn-Asp** | 0.002625 ± 0.001746 | 0.000178 ± 0.001136 | 0.000583 ± 0.001823 | 0.001 | 0.001 | 0.001 |
| **Freq of Asn-Glu** | 0.001438 ± 0.002279 | 0.000116 ± 0.000679 | 0.000448 ± 0.001547 | 0.001 | 0.001 | 0.001 |
| **Freq of Asn-Phe** | 0.000875 ± 0.001147 | 0.000112 ± 0.000641 | 0.000203 ± 0.000765 | 0.001 | NS | 0.001 |
| **Freq of Asn-Gly** | 0.003313 ± 0.002213 | 0.000229 ± 0.001369 | 0.000559± 0.001784 | 0.001 | NS | 0.001 |
| **Freq of Asn-His** | 0.000938 ± 0.000998 | 0.000109 ± 0.000703 | 0.000210± 0.000794 | 0.001 | NS | 0.001 |
| **Freq of Asn-Ile** | 0.002125 ± 0.001746 | 0.000205 ±0.001174 | 0.000369 ± 0.001325 | 0.001 | NS | 0.01 |
| **Freq of Asn-Lys** | 0.002688 ± 0.002496 | 0.000140 ± 0.000820 | 0.000334 ± 0.001298 | 0.001 | 0.001 | 0.01 |
| **Freq of Asn-Leu** | 0.004063 ± 0.002768 | 0.000256 ± 0.001365 | 0.000617 ±0.001849 | 0.001 | NS | 0.001 |
| **Freq of Asn-Met** | 0.001250 ± 0.001915 | 0.000101 ± 0.000640 | 0.000241 ± 0.000928 | 0.001 | NS | 0.001 |
| **Freq of Asn-Asn** | 0.000438 ± 0.000892 | 0.000244 ± 0.001512 | 0.000310 ± 0.001474 | NS | NS | 0.05 |
| **Freq of Asn-Pro** | 0.002500 ±0.001826 | 0.000178 ± 0.000912 | 0.000407 ± 0.001217 | 0.001 | NS | 0.001 |
| **Freq of Asn-Gln** | 0.001625 ± 0.001996 | 0.0000698 ± 0.000494 | 0.000272 ± 0.000962 | 0.001 | NS | 0.001 |
| **Freq of Asn-Arg** | 0.001750 ± 0.003715 | 0.0000969 ± 0.000580 | 0.000231± 0.001134 | 0.001 | NS | 0.01 |
| **Freq of Asn-Ser** | 0.002813 ± 0.002639 | 0.000318 ± 0.001869 | 0.000562 ± 0.002024 | 0.001 | NS | 0.001 |
| **Freq of Asn-Thr** | 0.002875 ± 0.003481 | 0.000198 ± 0.001192 | 0.000583 ± 0.001951 | 0.001 | 0.05 | 0.001 |
| **Freq of Asn-Val** | 0.001875 ± 0.003538 | 0.000136 ± 0.000804 | 0.000555 ± 0.001871 | 0.001 | 0.001 | 0.001 |
| **Freq of Asn-Trp** | 0.000250 ± 0.000447 | 0.00000388 ± 0.0000623 | 0.0000793 ± 0.000468 | 0.05 | 0.001 | 0.001 |
| **Freq of Asn-Tyr** | 0.000813 ± 0.001682 | 0.0000736 ± 0.000557 | 0.000255 ± 0.001064 | 0.01 | 0.001 | 0.001 |
| **Freq of Pro-Ala** | 0.003125 ± 0.002941 | 0.000178 ± 0.000916 | 0.000490 ± 0.001472 | 0.001 | NS | 0.001 |
| **Freq of Pro-Cys** | 0.000000000±0.000000000 | 0.0000465 ± 0.000456 | 0.0000690± 0.000487 | NS | 0.01 | 0.001 |
| **Freq of Pro-Asp** | 0.000625 ± 0.001025 | 0.0000930 ± 0.000716 | 0.000355± 0.001437 | NS | 0.001 | 0.001 |
| **Freq of Pro-Glu** | 0.002125 ± 0.001857 | 0.000202 ± 0.001173 | 0.000445 ± 0.001436 | 0.001 | NS | 0.001 |
| **Freq of Pro-Phe** | 0.001125 ± 0.001668 | 0.000112 ± 0.000763 | 0.000162 ± 0.000847 | 0.001 | 0.001 | NS |
| **Freq of Pro-Gly** | 0.001250 ± 0.001528 | 0.000112 ±0.000705 | 0.000579 ± 0.001914 | 0.001 | 0.001 | 0.001 |
| **Freq of Pro-His** | 0.001313 ± 0.001537 | 0.0000504 ± 0.000493 | 0.000117± 0.000650 | 0.001 | 0.001 | NS |
| **Freq of Pro-Ile** | 0.000813 ± 0.001109 | 0.000155 ± 0.000855 | 0.000190 ± 0.000862 | 0.01 | NS | NS |
| **Freq of Pro-Lys** | 0.001688 ± 0.001957 | 0.0000775 ± 0.000631 | 0.000231 ± 0.000940 | 0.001 | NS | 0.001 |
| **Freq of Pro-Leu** | 0.003500 ± 0.001826 | 0.000209 ± 0.001103 | 0.000414 ± 0.001387 | 0.001 | 0.001 | NS |
| **Freq of Pro-Met** | 0.000438 ± 0.000727 | 0.0000814 ± 0.000658 | 0.000138 ± 0.000736 | NS | NS | 0.001 |
| **Freq of Pro-Asn** | 0.002250 ± 0.002769 | 0.000132 ± 0.000763 | 0.000503 ± 0.001859 | 0.001 | 0.001 | 0.001 |
| **Freq of Pro-Pro** | 0.00369 ± 0.00491 | 0.000167 ± 0.001091 | 0.000372 ± 0.001729 | 0.001 | 0.001 | NS |
| **Freq of Pro-Gln** | 0.001313 ± 0.001250 | 0.000147 ± 0.000842 | 0.000407 ± 0.001262 | 0.001 | 0.001 | 0.001 |
| **Freq of Pro-Arg** | 0.001063 ± 0.001063 | 0.000132 ± 0.000732 | 0.000238 ± 0.000837 | 0.001 | NS | 0.001 |
| **Freq of Pro-Ser** | 0.004750 ± 0.003416 | 0.000248 ± 0.001370 | 0.000717 ± 0.002080 | 0.001 | NS | 0.001 |
| **Freq of Pro-Thr** | 0.002938 ± 0.002594 | 0.000112 ± 0.000705 | 0.000397± 0.001258 | 0.001 | NS | 0.001 |
| **Freq of Pro-Val** | 0.004813 ± 0.003868 | 0.000171 ± 0.001070 | 0.000541± 0.001786 | 0.001 | 0.001 | 0.001 |
| **Freq of Pro-Trp** | 0.000188 ± 0.000750 | 0.0000116 ± 0.000139 | 0.0000759± 0.000383 | NS | 0.001 | 0.001 |
| **Freq of Pro-Tyr** | 0.002438 ± 0.001711 | 0.0000426 ± 0.000283 | 0.000248 ± 0.000803 | 0.001 | 0.001 | 0.001 |
| **Freq of Gln-Ala** | 0.002625 ± 0.002705 | 0.000151 ± 0.000876 | 0.000431± 0.001327 | 0.001 | NS | 0.001 |
| **Freq of Gln-Cys** | 0.000250 ± 0.000775 | 0.0000465 ± 0.000481 | 0.0000552 ± 0.000489 | NS | NS | NS |
| **Freq of Gln-Asp** | 0.001438 ± 0.001672 | 0.0000891 ±0.000554 | 0.000179 ± 0.000736 | 0.001 | 0.001 | NS |
| **Freq of Gln-Glu** | 0.002188 ± 0.001642 | 0.000163 ± 0.001038 | 0.000369± 0.001336 | 0.001 | NS | 0.001 |
| **Freq of Gln-Phe** | 0.001563 ± 0.002581 | 0.0000853 ± 0.000630 | 0.000238± 0.001016 | 0.001 | NS | 0.001 |
| **Freq of Gln-Gly** | 0.001750 ± 0.001844 | 0.0000891 ± 0.000582 | 0.000497± 0.001643 | 0.001 | 0.001 | 0.001 |
| **Freq of Gln-His** | 0.000563 ± 0.000892 | 0.0000620 ±0.000495 | 0.000141 ± 0.000653 | 0.01 | NS | 0.001 |
| **Freq of Gln-Ile** | 0.002188 ± 0.001870 | 0.000120± 0.000747 | 0.000262 ± 0.001005 | 0.001 | 0.001 | NS |
| **Freq of Gln-Lys** | 0.001875 ± 0.001746 | 0.000109 ± 0.000751 | 0.000228 ± 0.000961 | 0.001 | 0.001 | NS |
| **Freq of Gln-Leu** | 0.005000 ± 0.003098 | 0.000178 ± 0.001170 | 0.000524± 0.001761 | 0.001 | 0.001 | 0.001 |
| **Freq of Gln-Met** | 0.001375 ±0.001668 | 0.0000233 ±0.000215 | 0.0000966 ± 0.000530 | 0.001 | 0.001 | NS |
| **Freq of Gln-Asn** | 0.001188 ± 0.001276 | 0.000101 ±0.000609 | 0.000286 ± 0.000917 | 0.001 | 0.001 | 0.001 |
| **Freq of Gln-Pro** | 0.000750 ± 0.001844 | 0.000124 ±0.000799 | 0.000179 ± 0.000897 | 0.05 | NS | NS |
| **Freq of Gln-Gln** | 0.001313 ± 0.001580 | 0.000132 ± 0.001005 | 0.000421 ± 0.001463 | 0.001 | 0.001 | 0.001 |
| **Freq of Gln-Arg** | 0.001875 ± 0.001962 | 0.000116 ±0.000918 | 0.000248 ± 0.001107 | 0.001 | 0.01 | 0.05 |
| **Freq of Gln-Ser** | 0.002563 ± 0.002308 | 0.000194 ±0.001077 | 0.000376 ± 0.001347 | 0.001 | 0.01 | 0.01 |
| **Freq of Gln-Thr** | 0.001750 ± 0.001732 | 0.0000736 ±0.000520 | 0.000231± 0.000839 | 0.001 | NS | 0.001 |
| **Freq of Gln-Val** | 0.002250 ± 0.001949 | 0.0000853 ±0.000579 | 0.000434 ± 0.001423 | 0.001 | 0.001 | 0.001 |
| **Freq of Gln-Trp** | 0.000000000±0.000000000 | 0.0000194 ± 0.000224 | 0.000141 ± 0.000719 | NS | 0.001 | 0.001 |
| **Freq of Gln-Tyr** | 0.000813 ± 0.001167 | 0.000105 ±0.000661 | 0.000359 ± 0.001260 | 0.01 | 0.001 | 0.001 |
| **Freq of Arg-Ala** | 0.002313 ±0.001815 | 0.0000853 ± 0.000530 | 0.000341± 0.001061 | 0.001 | NS | 0.001 |
| **Freq of Arg-Cys** | 0.000750 ±0.000931 | 0.0000310 ± 0.000277 | 0.0000690± 0.000375 | 0.001 | 0.001 | NS |
| **Freq of Arg-Asp** | 0.001375 ± 0.001784 | 0.000136 ± 0.000799 | 0.000383 ± 0.001174 | 0.001 | 0.001 | 0.001 |
| **Freq of Arg-Glu** | 0.002063 ± 0.002112 | 0.000105 ± 0.000618 | 0.000321 ± 0.001044 | 0.001 | NS | 0.001 |
| **Freq of Arg-Phe** | 0.001500 ± 0.001673 | 0.0000930 ±0.000688 | 0.000193 ± 0.000847 | 0.001 | 0.001 | NS |
| **Freq of Arg-Gly** | 0.003375 ± 0.002363 | 0.000105± 0.000913 | 0.000476 ± 0.001666 | 0.001 | NS | 0.001 |
| **Freq of Arg-His** | 0.000625 ± 0.000957 | 0.0000930 ±0.000610 | 0.000117 ± 0.000628 | 0.01 | 0.01 | NS |
| **Freq of Arg-Ile** | 0.004063 ± 0.002112 | 0.000140 ±0.000996 | 0.000428 ± 0.001573 | 0.001 | 0.001 | 0.001 |
| **Freq of Arg-Lys** | 0.002500 ± 0.001826 | 0.000302 ±0.001423 | 0.000462 ± 0.001516 | 0.001 | 0.01 | NS |
| **Freq of Arg-Leu** | 0.00594 ± 0.00673 | 0.000322 ± 0.001655 | 0.000710 ± 0.002595 | 0.001 | 0.001 | 0.05 |
| **Freq of Arg-Met** | 0.000875 ± 0.001088 | 0.0000659 ± 0.000506 | 0.000107 ± 0.000569 | 0.001 | 0.001 | NS |
| **Freq of Arg-Asn** | 0.00313 ± 0.00426 | 0.000128 ±0.000740 | 0.000390 ± 0.001454 | 0.001 | NS | 0.001 |
| **Freq of Arg-Pro** | 0.001750 ± 0.002176 | 0.000143 ± 0.001343 | 0.000224 ± 0.001410 | 0.001 | 0.001 | NS |
| **Freq of Arg-Gln** | 0.001813 ± 0.001834 | 0.000128 ± 0.000923 | 0.000269± 0.001080 | 0.001 | NS | 0.01 |
| **Freq of Arg-Arg** | 0.001875 ± 0.001821 | 0.000271 ±0.001514 | 0.000469 ± 0.001620 | 0.001 | NS | 0.001 |
| **Freq of Arg-Ser** | 0.003375 ± 0.002500 | 0.000229 ±0.001225 | 0.000690 ± 0.002012 | 0.001 | 0.001 | 0.001 |
| **Freq of Arg-Thr** | 0.002688 ± 0.001815 | 0.000143 ± 0.000793 | 0.000410 ± 0.001286 | 0.001 | NS | 0.001 |
| **Freq of Arg-Val** | 0.002813 ± 0.002373 | 0.000163 ±0.001083 | 0.000431 ± 0.001437 | 0.001 | NS | 0.001 |
| **Freq of Arg-Trp** | 0.000250 ± 0.000447 | 0.0000581 ± 0.000565 | 0.0000793 ± 0.000568 | NS | NS | NS |
| **Freq of Arg-Tyr** | 0.001063 ± 0.001063 | 0.0000543 ± 0.000349 | 0.000121 ± 0.000495 | 0.001 | 0.001 | NS |
| **Freq of Ser-Ala** | 0.006063 ± 0.003172 | 0.000252 ±0.001418 | 0.001162 ± 0.003418 | 0.001 | 0.001 | 0.001 |
| **Freq of Ser-Cys** | 0.000688 ± 0.001537 | 0.0000155 ± 0.000124 | 0.0000586 ± 0.000416 | 0.001 | 0.001 | NS |
| **Freq of Ser-Asp** | 0.004313 ± 0.003381 | 0.000159 ±0.000922 | 0.000807 ± 0.002290 | 0.001 | 0.001 | 0.001 |
| **Freq of Ser-Glu** | 0.004125 ±0.002446 | 0.000329 ±0.001604 | 0.000745 ± 0.002059 | 0.001 | NS | 0.001 |
| **Freq of Ser-Phe** | 0.004188± 0.002007 | 0.000143 ± 0.000841 | 0.000441 ± 0.001381 | 0.001 | 0.001 | 0.001 |
| **Freq of Ser-Gly** | 0.00656 ± 0.00596 | 0.000337± 0.001650 | 0.001266 ± 0.003564 | 0.001 | 0.001 | 0.001 |
| **Freq of Ser-His** | 0.003750 ±0.003152 | 0.000151 ± 0.001023 | 0.000383 ± 0.001477 | 0.001 | 0.001 | NS |
| **Freq of Ser-Ile** | 0.004000 ± 0.002733 | 0.000209 ±0.001085 | 0.000548± 0.001621 | 0.001 | 0.01 | 0.001 |
| **Freq of Ser-Lys** | 0.004875 ± 0.002473 | 0.000353± 0.001761 | 0.000734± 0.002208 | 0.001 | 0.01 | 0.001 |
| **Freq of Ser-Leu** | 0.00900 ± 0.00575 | 0.000527 ± 0.002464 | 0.001410± 0.003745 | 0.001 | NS | 0.001 |
| **Freq of Ser-Met** | 0.001688 ±0.001352 | 0.0000814 ±0.000527 | 0.000317 ± 0.001030 | 0.001 | 0.001 | 0.001 |
| **Freq of Ser-Asn** | 0.002313 ±0.002056 | 0.000368 ± 0.001949 | 0.000814 ± 0.002620 | 0.01 | 0.001 | 0.001 |
| **Freq of Ser-Pro** | 0.002688 ±0.002938 | 0.000236 ± 0.001423 | 0.000462 ± 0.001703 | 0.001 | NS | 0.001 |
| **Freq of Ser-Gln** | 0.001563 ±0.001459 | 0.000205± 0.001081 | 0.000490 ± 0.001496 | 0.001 | 0.001 | 0.001 |
| **Freq of Ser-Arg** | 0.003250 ±0.001612 | 0.000155 ±0.000886 | 0.000448 ± 0.001414 | 0.001 | NS | 0.001 |
| **Freq of Ser-Ser** | 0.01050 ± 0.00608 | 0.000597 ± 0.003419 | 0.001514 ± 0.004535 | 0.001 | 0.05 | 0.001 |
| **Freq of Ser-Thr** | 0.00631 ± 0.00474 | 0.000302 ± 0.001453 | 0.000962 ± 0.002697 | 0.001 | NS | 0.001 |
| **Freq of Ser-Val** | 0.006063 ± 0.002294 | 0.000287 ±0.001448 | 0.001169 ± 0.003144 | 0.001 | 0.001 | 0.001 |
| **Freq of Ser-Trp** | 0.001813 ± 0.001721 | 0.0000543 ± 0.000541 | 0.000203 ± 0.000834 | 0.001 | 0.05 | 0.001 |
| **Freq of Ser-Tyr** | 0.002313 ± 0.001852 | 0.000128 ±0.000791 | 0.000579 ± 0.001943 | 0.001 | 0.001 | 0.001 |
| **Freq of Thr-Ala** | 0.004375 ± 0.003263 | 0.000151 ± 0.001046 | 0.000769 ± 0.002269 | 0.001 | 0.001 | 0.001 |
| **Freq of Thr-Cys** | 0.000188 ± 0.000750 | 0.0000465 ±0.000392 | 0.0000931 ± 0.000494 | NS | 0.01 | 0.001 |
| **Freq of Thr-Asp** | 0.002563 ± 0.001931 | 0.000112 ±0.000705 | 0.000517 ± 0.001641 | 0.001 | 0.001 | 0.001 |
| **Freq of Thr-Glu** | 0.001438 ± 0.001750 | 0.000163 ± 0.000835 | 0.000366 ± 0.001184 | 0.001 | 0.01 | 0.001 |
| **Freq of Thr-Phe** | 0.002750 ± 0.001693 | 0.0000659 ± 0.000394 | 0.000317 ± 0.000975 | 0.001 | 0.01 | 0.001 |
| **Freq of Thr-Gly** | 0.00525 ± 0.00477 | 0.000163 ± 0.001185 | 0.000738 ± 0.002572 | 0.001 | NS | 0.001 |
| **Freq of Thr-His** | 0.002375 ± 0.001857 | 0.0000233 ± 0.000232 | 0.000269± 0.000878 | 0.001 | NS | 0.001 |
| **Freq of Thr-Ile** | 0.002625 ± 0.001708 | 0.000159 ± 0.000999 | 0.000507 ± 0.001585 | 0.001 | 0.01 | 0.001 |
| **Freq of Thr-Lys** | 0.00544 ± 0.00666 | 0.000190 ± 0.001215 | 0.000566 ± 0.002348 | 0.001 | 0.001 | 0.01 |
| **Freq of Thr-Leu** | 0.00731 ± 0.00401 | 0.000295 ±0.001742 | 0.001010 ± 0.002981 | 0.001 | NS | 0.001 |
| **Freq of Thr-Met** | 0.000438 ± 0.001031 | 0.0000194 ±0.000164 | 0.000124± 0.000538 | 0.001 | 0.001 | 0.001 |
| **Freq of Thr-Asn** | 0.000688 ± 0.000946 | 0.000128 ±0.000761 | 0.000486± 0.001743 | NS | 0.001 | 0.001 |
| **Freq of Thr-Pro** | 0.001188 ± 0.001834 | 0.000128 ± 0.000674 | 0.000352 ± 0.001188 | 0.001 | 0.001 | 0.001 |
| **Freq of Thr-Gln** | 0.001875 ±0.001586 | 0.0000659 ± 0.000449 | 0.000300 ± 0.001050 | 0.001 | NS | 0.001 |
| **Freq of Thr-Arg** | 0.002375 ± 0.001821 | 0.000140 ±0.000848 | 0.000334 ± 0.001123 | 0.001 | 0.05 | 0.001 |
| **Freq of Thr-Ser** | 0.003563 ± 0.002449 | 0.000291 ±0.001537 | 0.000817 ± 0.002367 | 0.001 | 0.001 | 0.001 |
| **Freq of Thr-Thr** | 0.002563 ± 0.001896 | 0.000205 ± 0.001219 | 0.000672 ± 0.001988 | 0.001 | 0.001 | 0.001 |
| **Freq of Thr-Val** | 0.005313 ± 0.001815 | 0.000140 ±0.000875 | 0.000666 ± 0.001901 | 0.001 | NS | 0.001 |
| **Freq of Thr-Trp** | 0.001000 ± 0.001751 | 0.0000310 ± 0.000383 | 0.000152 ± 0.000756 | 0.001 | NS | 0.001 |
| **Freq of Thr-Tyr** | 0.001375 ± 0.001360 | 0.0000969 ± 0.000607 | 0.000300 ± 0.000954 | 0.001 | 0.001 | 0.001 |
| **Freq of Val-Ala** | 0.005750 ± 0.003751 | 0.000205 ± 0.001320 | 0.001014 ± 0.002875 | 0.001 | 0.001 | 0.001 |
| **Freq of Val-Cys** | 0.000750 ± 0.001183 | 0.0000388 ±0.000505 | 0.0000897 ± 0.000593 | 0.001 | 0.05 | NS |
| **Freq of Val-Asp** | 0.003625 ± 0.003442 | 0.000155 ± 0.000974 | 0.000828 ± 0.002481 | 0.001 | 0.001 | 0.001 |
| **Freq of Val-Glu** | 0.003750 ± 0.002017 | 0.000171 ± 0.000967 | 0.000731 ± 0.002250 | 0.001 | 0.001 | 0.001 |
| **Freq of Val-Phe** | 0.003625 ± 0.002446 | 0.0000969 ± 0.000567 | 0.000300 ± 0.001124 | 0.001 | 0.001 | NS |
| **Freq of Val-Gly** | 0.003438 ± 0.001788 | 0.000186 ± 0.001252 | 0.000752± 0.002177 | 0.001 | 0.001 | 0.001 |
| **Freq of Val-His** | 0.002000 ± 0.001862 | 0.0000620 ±0.000561 | 0.000238± 0.000924 | 0.001 | NS | 0.001 |
| **Freq of Val-Ile** | 0.004188 ± 0.002509 | 0.000105 ± 0.000631 | 0.000490± 0.001489 | 0.001 | 0.01 | 0.001 |
| **Freq of Val-Lys** | 0.006438 ± 0.003346 | 0.000163 ±0.000844 | 0.000652 ± 0.002098 | 0.001 | 0.001 | 0.001 |
| **Freq of Val-Leu** | 0.00956 ± 0.00588 | 0.000267 ± 0.001589 | 0.001062± 0.003128 | 0.001 | 0.001 | 0.001 |
| **Freq of Val-Met** | 0.001750 ± 0.001483 | 0.0000581 ± 0.000433 | 0.000203± 0.000760 | 0.001 | 0.01 | 0.001 |
| **Freq of Val-Asn** | 0.001625 ± 0.001928 | 0.000132 ± 0.000737 | 0.000445 ± 0.001354 | 0.001 | 0.001 | 0.001 |
| **Freq of Val-Pro** | 0.002500 ± 0.002280 | 0.0000853 ± 0.000579 | 0.000303 ± 0.001028 | 0.001 | 0.01 | 0.001 |
| **Freq of Val-Gln** | 0.004063 ± 0.001948 | 0.000112 ± 0.000912 | 0.000379± 0.001360 | 0.001 | 0.001 | 0.01 |
| **Freq of Val-Arg** | 0.002750 ± 0.002887 | 0.000109 ±0.000746 | 0.000362 ± 0.001317 | 0.001 | NS | 0.001 |
| **Freq of Val-Ser** | 0.00719 ± 0.00409 | 0.000298 ± 0.001558 | 0.001314 ± 0.003536 | 0.001 | 0.001 | 0.001 |
| **Freq of Val-Thr** | 0.003500 ± 0.003967 | 0.000159 ± 0.000959 | 0.000793 ± 0.002453 | 0.001 | 0.001 | 0.001 |
| **Freq of Val-Val** | 0.005375 ± 0.002419 | 0.000163 ± 0.001035 | 0.000907 ± 0.002567 | 0.001 | 0.001 | 0.001 |
| **Freq of Val-Trp** | 0.001000 ± 0.001713 | 0.0000271 ± 0.000378 | 0.000107± 0.000659 | 0.001 | NS | 0.01 |
| **Freq of Val-Tyr** | 0.000875 ± 0.001258 | 0.0000853 ± 0.000537 | 0.000159± 0.000698 | 0.001 | NS | 0.01 |
| **Freq of Trp-Ala** | 0.001438 ± 0.001263 | 0.0000736 ± 0.000763 | 0.000207 ± 0.000907 | 0.001 | NS | 0.001 |
| **Freq of Trp-Cys** | 0.000313 ± 0.001250 | 0.00000388 ± 0.0000623 | 0.0000276 ± 0.000310 | 0.001 | NS | NS |
| **Freq of Trp-Asp** | 0.000625 ± 0.001025 | 0.0000388 ± 0.000372 | 0.000186 ± 0.000706 | 0.001 | 0.001 | 0.001 |
| **Freq of Trp-Glu** | 0.001188 ± 0.001109 | 0.0000388 ± 0.000372 | 0.000203 ± 0.000809 | 0.001 | 0.01 | 0.001 |
| **Freq of Trp-Phe** | 0.000000000±0.000000000 | 0.0000349 ± 0.000388 | 0.0000310 ± 0.000366 | NS | NS | NS |
| **Freq of Trp-Gly** | 0.000563 ± 0.000629 | 0.00000388 ± 0.0000623 | 0.0000828 ± 0.000390 | 0.001 | 0.05 | 0.001 |
| **Freq of Trp-His** | 0.0000625 ± 0.000250 | 0.0000116 ± 0.000107 | 0.0000138± 0.000117 | NS | NS | NS |
| **Freq of Trp-Ile** | 0.000000000±0.000000000 | 0.000000000±0.000000000 | 0.00000690 ± 0.000117 | 0 | 0.001 | 0.001 |
| **Freq of Trp-Lys** | 0.002625 ± 0.001893 | 0.0000349 ± 0.000388 | 0.000176 ± 0.000819 | 0.001 | 0.001 | NS |
| **Freq of Trp-Leu** | 0.001000 ± 0.000966 | 0.0000310 ± 0.000231 | 0.0000828 ± 0.000381 | 0.001 | 0.001 | NS |
| **Freq of Trp-Met** | 0.0000625 ± 0.000250 | 0.0000194 ± 0.000206 | 0.0000207 ± 0.000203 | NS | NS | NS |
| **Freq of Trp-Asn** | 0.000250 ± 0.000447 | 0.0000620 ± 0.000651 | 0.000103 ± 0.000673 | NS | NS | 0.01 |
| **Freq of Trp-Pro** | 0.001313 ± 0.001302 | 0.000000000±0.000000000 | 0.0000724 ± 0.000422 | 0.001 | 0.001 | NS |
| **Freq of Trp-Gln** | 0.000125 ± 0.000342 | 0.0000194 ± 0.000206 | 0.0000241 ± 0.000211 | NS | NS | NS |
| **Freq of Trp-Arg** | 0.001188 ± 0.001682 | 0.0000349 ± 0.000388 | 0.000117 ± 0.000639 | 0.001 | 0.001 | NS |
| **Freq of Trp-Ser** | 0.001563 ± 0.001711 | 0.0000891 ± 0.000663 | 0.000262 ± 0.000927 | 0.001 | NS | 0.001 |
| **Freq of Trp-Thr** | 0.0000625 ± 0.000250 | 0.0000233 ± 0.000374 | 0.000107 ± 0.000644 | NS | 0.001 | 0.001 |
| **Freq of Trp-Val** | 0.000250 ± 0.000775 | 0.00000388 ± 0.0000623 | 0.0000241 ± 0.000211 | 0.001 | NS | NS |
| **Freq of Trp-Trp** | 0.000000000±0.000000000 | 0.0000116 ± 0.000107 | 0.0000310 ± 0.000268 | 0.01 | 0.01 | 0.01 |
| **Freq of Trp-Tyr** | 0.000125 ± 0.000342 | 0.00000775 ± 0.0000879 | 0.0000138 ± 0.000117 | 0.001 | 0.01 | NS |
| **Freq of Tyr-Ala** | 0.001063 ± 0.001063 | 0.0000698 ± 0.000532 | 0.000424 ± 0.001475 | 0.001 | 0.001 | 0.001 |
| **Freq of Tyr-Cys** | 0.000250 ± 0.000775 | 0.0000388 ± 0.000303 | 0.0000483± 0.000340 | 0.05 | NS | NS |
| **Freq of Tyr-Asp** | 0.002000 ± 0.002098 | 0.000112 ± 0.000641 | 0.000503± 0.001683 | 0.001 | 0.001 | 0.001 |
| **Freq of Tyr-Glu** | 0.001813 ± 0.002198 | 0.0000891 ± 0.000525 | 0.000345 ± 0.001140 | 0.001 | 0.001 | 0.001 |
| **Freq of Tyr-Phe** | 0.002063 ± 0.001652 | 0.0000698 ± 0.000554 | 0.000176 ± 0.000789 | 0.001 | 0.001 | NS |
| **Freq of Tyr-Gly** | 0.001875 ± 0.001708 | 0.0000969 ± 0.000594 | 0.000379 ± 0.001286 | 0.001 | 0.001 | 0.001 |
| **Freq of Tyr-His** | 0.0000625 ± 0.000250 | 0.0000233 ± 0.000175 | 0.0000379 ± 0.000240 | NS | NS | 0.001 |
| **Freq of Tyr-Ile** | 0.002500 ± 0.001966 | 0.0000969 ± 0.000644 | 0.000252 ± 0.000957 | 0.001 | 0.001 | NS |
| **Freq of Tyr-Lys** | 0.002500 ± 0.001713 | 0.0000969 ± 0.000619 | 0.000252 ± 0.000916 | 0.001 | 0.001 | NS |
| **Freq of Tyr-Leu** | 0.003500 ±0.003286 | 0.0000969 ± 0.000662 | 0.000376± 0.001321 | 0.001 | 0.001 | 0.001 |
| **Freq of Tyr-Met** | 0.000125 ±0.000342 | 0.0000155 ± 0.000152 | 0.0000207± 0.000165 | 0.05 | NS | NS |
| **Freq of Tyr-Asn** | 0.000375 ± 0.000806 | 0.0000465 ± 0.000338 | 0.000152 ± 0.000659 | NS | 0.001 | 0.001 |
| **Freq of Tyr-Pro** | 0.001500 ± 0.001932 | 0.0000504 ± 0.000386 | 0.000328 ± 0.001197 | 0.001 | 0.001 | 0.001 |
| **Freq of Tyr-Gln** | 0.001375 ± 0.001668 | 0.0000853 ± 0.000544 | 0.000200 ± 0.000807 | 0.001 | NS | 0.001 |
| **Freq of Tyr-Arg** | 0.001188 ± 0.001276 | 0.0000969 ± 0.000883 | 0.000179 ± 0.000942 | 0.001 | NS | NS |
| **Freq of Tyr-Ser** | 0.001438 ± 0.001711 | 0.000151 ± 0.001042 | 0.000379 ± 0.001460 | 0.001 | 0.01 | 0.001 |
| **Freq of Tyr-Thr** | 0.002438 ± 0.001750 | 0.0000891 ± 0.000633 | 0.000366 ± 0.001136 | 0.001 | NS | 0.001 |
| **Freq of Tyr-Val** | 0.002500 ± 0.002608 | 0.0000543 ± 0.000370 | 0.000345 ± 0.001205 | 0.001 | NS | 0.001 |
| **Freq of Tyr-Trp** | 0.000500 ± 0.000516 | 0.00000775 ± 0.0000879 | 0.0000345 ± 0.000183 | 0.001 | 0.001 | NS |
| **Freq of Tyr-Tyr** | 0.001125 ± 0.001025 | 0.0000698 ± 0.000510 | 0.000131± 0.000591 | 0.001 | 0.001 | NS |
